# Supplementary material for: Ultrastrong to nearly deep-strong magnon-magnon coupling with a high degree of freedom in synthetic antiferromagnets
Source: Nat Commun. 2024 Mar 7;15:2077. doi: 10.1038/s41467-024-46474-7 (PMC10920873; doi:10.1038/s41467-024-46474-7)
Supplement: Supplementary file 1 — Supplementary Information [file 41467_2024_46474_MOESM1_ESM.pdf]

Supplementary Materials for

Ultrastrong to nearly deep-strong magnon-magnon coupling with a high degree of freedom in synthetic antiferromagnets

Yuqiang Wang[1,2], Yu Zhang[3], Chaozhong Li[4], Jinwu Wei[4], Bin He[1,2], Hongjun Xu[1,5], Jihao Xia[1,2], Xuming Luo[1,2], Jiahui Li[1,2], Jing Dong[1,5], Wenqing He[1,2], Zhengren Yan[1,2], Wenlong Yang[1,2], Fusheng Ma[3]*, Guozhi Chai[4], Peng Yan[6], Caihua Wan[1,2], Xiufeng Han[1,2,5], Guoqiang Yu[1,2,5]*

[1]Beijing National Laboratory for Condensed Matter Physics, Institute of Physics, Chinese Academy of Sciences, Beijing 100190, China

[2]Center of Materials Science and Optoelectronics Engineering, University of Chinese Academy of Sciences, Beijing 100049, China

[3]Jiangsu Key Laboratory of Opto-Electronic Technology, School of Physics and Technology, Nanjing Normal University, Nanjing 210046, China

[4]Key Laboratory for Magnetism and Magnetic Materials of the Ministry of Education, Lanzhou University, Lanzhou 730000, China

[5]Songshan Lake Materials Laboratory, Dongguan, Guangdong 523808, China

[6]School of Electronic Science and Engineering and State Key Laboratory of Electronic Thin Films and Integrated Devices, University of Electronic Science and Technology of China, Chengdu 610054, China

*Corresponding authors.

Email: phymafs@njnu.edu.cn;

guoqiangyu@iphy.ac.cn

This PDF file includes:

Supplementary Text (Sections S1 to S16)
Figures S1 to S20
Tables S1 to S2

Supplementary Text

Section S1. Comparison of the coupling strength g_1 and the gap between the two branches

To illustrate the inaccuracy of taking half the gap as the coupling strength in our USC system, we compare the coupling strength g_1 derived from the quantum model with the gap. Figures S1a and S1b show two examples corresponding to the cases in Figs. 2b and 2a, respectively. In each plot, we calculate half the gap as a function of H_{k1} (red curve), and compare it with $g_1/2\pi$ (blue curve) at H_0. Deviation factor σ is defined as $|\mathrm{gap}/2 - g_1/2\pi(H_0)|/(g_1/2\pi(H_0))$, and we use grey to label the region with $\sigma < 0.1$. In Fig. S1a, $H_{k2} = 5$ kOe, the configuration is PMA SAF or T-Type depending on H_{k1}. We note that the grey region only exists in the vicinity of the symmetry PMA SAF case, which means half the gap is a good approximation of the coupling strength at H_0 only when the coupling is not strong. Near the boundary of the PMA SAF region, the σ can even larger than 0.5. This derivation mainly originates from the special spectral structure of PMA SAF. In the T-Type region, gap/2 is totally different from $g_1/2\pi(H_0)$, which indicates a complete failure of taking half the gap as the coupling strength. In Fig. S1b, $H_{k2} = -5$ kOe, the configuration is IP SAF or T-Type depending on H_{k1}. We first consider the IP SAF case. Different from PMA SAF case where gap/2 is smaller than $g_1/2\pi(H_0)$ and the grey region is small, in IP SAF case, gap/2 is larger than $g_1/2\pi(H_0)$ and the grey region is much larger than PMA SAF case. Still, near the boundary of the IP SAF region, the σ is larger than 0.1, which is mainly due to the effect of the counter-rotating term. When the configuration changes from IP SAF to T-Type, $g_1/2\pi(H_0)$ is discontinuous while gap/2 does not show this property. And similarly, in T-Type region, gap/2 is totally different from $g_1/2\pi(H_0)$. To illustrate the universality of the above results, gap/2 and $g_1/2\pi(H_0)$ as functions of H_{k1} and H_{k2} are shown in Figs. S1c and S1d, respectively, which show consistency with Figs. S1a and S1b.

Section S2. Method to identify the configurations of SAFs

We consider the configurations based on the simplified case. The coordinate system is defined the same as in Fig. 1c. At zero external magnetic field, Equation 2 in Methods can be rewritten as

$$\tilde{E} = -\frac{1}{2}H_{k1}\cos^2\theta_1 - \frac{1}{2}H_{k2}\cos^2\theta_2 + H_{ex}\cos(\theta_1+\theta_2) \tag{S-1}$$

where $\tilde{E} = E/\mu_0 dM_S$. In Eq. S-1, we assume that the equilibrium normalized magnetic moments $\mathbf{m}_1^{\mathbf{eq}}$ and $\mathbf{m}_2^{\mathbf{eq}}$ are always in *y*-*z* plane and θ_1 (θ_2) is defined as the angle at which $\mathbf{m_1}$ ($\mathbf{m_2}$) deviates clockwise (anticlockwise) from the +*z* (−*z*) axis. The equilibrium positions of θ_1 and θ_2 are marked as θ_1^{eq} and θ_2^{eq}, respectively, which should satisfy the following two relationships:

$$\left.\frac{\partial \tilde{E}}{\partial \theta_1}\right|_{\mathrm{eq}} = 0 \qquad \left.\frac{\partial \tilde{E}}{\partial \theta_2}\right|_{\mathrm{eq}} = 0 \tag{S-2}$$

and

$$\left.\frac{\partial^2 \tilde{E}}{\partial \theta_1^2}\right|_{\mathrm{eq}} > 0 \qquad \left.\frac{\partial^2 \tilde{E}}{\partial \theta_1^2}\frac{\partial^2 \tilde{E}}{\partial \theta_2^2} - \frac{\partial^2 \tilde{E}}{\partial \theta_2 \partial \theta_1}\frac{\partial^2 \tilde{E}}{\partial \theta_1 \partial \theta_2}\right|_{\mathrm{eq}} > 0 \tag{S-3}$$

For IP SAF, the magnetic moments of FM1 and FM2 are arranged antiparallel along the *y* axis, thus (θ_1^{eq}, θ_2^{eq}) is equal to (π/2, −π/2) or (−π/2, π/2). By substituting the above (θ_1^{eq}, θ_2^{eq}) into Eq. S-2 and Eq. S-3, the condition for IP SAF can be obtained:

$$H_{\mathrm{k1}} + H_{\mathrm{ex}} < 0 \qquad \left(H_{\mathrm{k1}} + H_{\mathrm{ex}}\right)\left(H_{\mathrm{k2}} + H_{\mathrm{ex}}\right) - H_{\mathrm{ex}}^2 > 0 \tag{S-4}$$

For PMA SAF, the magnetic moments of FM1 and FM2 are arranged antiparallel along the *z* axis, thus (θ_1^{eq}, θ_2^{eq}) is equal to (0, 0) or (π, π). Similarly, the condition for PMA SAF can be obtained:

$$H_{\mathrm{k1}} - H_{\mathrm{ex}} > 0 \qquad \left(H_{\mathrm{k1}} - H_{\mathrm{ex}}\right)\left(H_{\mathrm{k2}} - H_{\mathrm{ex}}\right) - H_{\mathrm{ex}}^2 > 0 \tag{S-5}$$

We note that for (H_{k1}, H_{k2}) which does not satisfy Eq. S-4 and Eq. S-5, $\mathbf{m}_1^{\mathbf{eq}}$ and $\mathbf{m}_2^{\mathbf{eq}}$ are canted, which means this case corresponds to T-Type.

Section S3. The expressions of the matrix elements $A_{\mathrm{ij}}^{\mathrm{k}}$

We first consider the simplified case. For IP SAF, A_{23}^1 and A_{41}^1 are equal to 0. The rest of the matrix elements have simple forms, which are displayed below:

$$\begin{aligned}
A_{12}^1 &= \frac{H_{k1} + H_{k2}}{2} \\
A_{14}^1 &= \frac{H_{k1} - H_{k2}}{2} \\
A_{21}^1 &= -2H_{ex} + \frac{H^2}{2H_{ex}} \\
A_{32}^1 &= \frac{H_{k1} - H_{k2}}{2} \\
A_{34}^1 &= \frac{H_{k1} + H_{k2}}{2} + 2H_{ex} \\
A_{43}^1 &= -\frac{H^2}{2H_{ex}}
\end{aligned} \qquad (H \le -2H_{ex})$$

and

$$\begin{aligned}
A_{12}^1 &= \frac{H_{k1} + H_{k2}}{2} - H - 2H_{ex} \\
A_{14}^1 &= \frac{H_{k1} - H_{k2}}{2} \\
A_{21}^1 &= 2H_{ex} + H \\
A_{32}^1 &= \frac{H_{k1} - H_{k2}}{2} \\
A_{34}^1 &= \frac{H_{k1} + H_{k2}}{2} - H \\
A_{43}^1 &= H
\end{aligned} \qquad (H > -2H_{ex})$$

For PMA SAF (T-Type), the matrix elements are displayed below:

$$A_{12}^2 = \frac{H + H_{ex}\sin\theta_2}{2\sin\theta_1} + \frac{H + H_{ex}\sin\theta_1}{2\sin\theta_2} - \frac{H_{k1}}{2}\sin^2\theta_1 - \frac{H_{k2}}{2}\sin^2\theta_2 - H_{ex}\cos(\theta_1 + \theta_2)$$

$$A_{14}^2 = \frac{H + H_{ex}\sin\theta_2}{2\sin\theta_1} - \frac{H + H_{ex}\sin\theta_1}{2\sin\theta_2} - \frac{H_{k1}}{2}\sin^2\theta_1 + \frac{H_{k2}}{2}\sin^2\theta_2$$

$$A_{21}^2 = -\frac{H + H_{ex}\sin\theta_2}{2\sin\theta_1} - \frac{H + H_{ex}\sin\theta_1}{2\sin\theta_2} - H_{ex}$$

$$A_{23}^2=-\frac{H+H_{\text{ex}}\sin\theta_2}{2\sin\theta_1}+\frac{H+H_{\text{ex}}\sin\theta_1}{2\sin\theta_2}$$

$$A_{32}^2=\frac{H+H_{\text{ex}}\sin\theta_2}{2\sin\theta_1}-\frac{H+H_{\text{ex}}\sin\theta_1}{2\sin\theta_2}-\frac{H_{\text{k1}}}{2}\sin^2\theta_1+\frac{H_{\text{k2}}}{2}\sin^2\theta_2$$

$$A_{34}^2=\frac{H+H_{\text{ex}}\sin\theta_2}{2\sin\theta_1}+\frac{H+H_{\text{ex}}\sin\theta_1}{2\sin\theta_2}-\frac{H_{\text{k1}}}{2}\sin^2\theta_1-\frac{H_{\text{k2}}}{2}\sin^2\theta_2+H_{\text{ex}}\cos\left(\theta_1+\theta_2\right)$$

$$A_{41}^2=-\frac{H+H_{\text{ex}}\sin\theta_2}{2\sin\theta_1}+\frac{H+H_{\text{ex}}\sin\theta_1}{2\sin\theta_2}$$

$$A_{43}^2=-\frac{H+H_{\text{ex}}\sin\theta_2}{2\sin\theta_1}-\frac{H+H_{\text{ex}}\sin\theta_1}{2\sin\theta_2}+H_{\text{ex}}$$

where θ_1 and θ_2 are obtained numerically from the following equations:

$$\begin{aligned}&H\cos\theta_1+H_{\text{ex}}\sin\left(\theta_1+\theta_2\right)-H_{\text{k1}}\cos\theta_1\sin\theta_1=0\\&H\cos\theta_2+H_{\text{ex}}\sin\left(\theta_1+\theta_2\right)-H_{\text{k2}}\cos\theta_2\sin\theta_2=0\end{aligned}\qquad\text{(S-6)}$$

We then consider the complete case. In this case, $\theta_{1(2)}$ and $\varphi_{1(2)}$ are all derived from numerical calculation. For IP SAF, the matrix elements are displayed below:

$$A_{12}^3=-\frac{I_1+I_2}{2}+\frac{H_{\text{k1}}}{2}+\frac{H_{\text{k2}}}{2}-\frac{H_{\text{ex1}}^{(1)}+H_{\text{ex1}}^{(2)}}{2}+\left(H_{\text{ex2}}^{(1)}+H_{\text{ex2}}^{(2)}\right)\cos\left(\varphi_1+\varphi_2\right)$$

$$A_{14}^3=-\frac{I_1-I_2}{2}+\frac{H_{\text{k1}}}{2}-\frac{H_{\text{k2}}}{2}+\frac{H_{\text{ex1}}^{(1)}-H_{\text{ex1}}^{(2)}}{2}-\left(H_{\text{ex2}}^{(1)}-H_{\text{ex2}}^{(2)}\right)\cos\left(\varphi_1+\varphi_2\right)$$

$$\begin{aligned}A_{21}^3=&\frac{I_1+I_2}{2}-\frac{H_{\text{ex1}}^{(1)}+H_{\text{ex1}}^{(2)}}{2}\cos\left(\varphi_1+\varphi_2\right)+\left(H_{\text{ex2}}^{(1)}+H_{\text{ex2}}^{(2)}\right)\cos^2\left(\varphi_1+\varphi_2\right)\\&-2\left(H_{\text{ex2}}^{(1)}+H_{\text{ex2}}^{(2)}\right)\sin^2\left(\varphi_1+\varphi_2\right)\end{aligned}$$

$$A_{23}^3=\frac{I_1-I_2}{2}+\frac{H_{\text{ex1}}^{(1)}-H_{\text{ex1}}^{(2)}}{2}\cos\left(\varphi_1+\varphi_2\right)-\left(H_{\text{ex2}}^{(1)}-H_{\text{ex2}}^{(2)}\right)\cos^2\left(\varphi_1+\varphi_2\right)$$

$$A_{32}^3=-\frac{I_1-I_2}{2}+\frac{H_{\text{k1}}}{2}-\frac{H_{\text{k2}}}{2}-\frac{H_{\text{ex1}}^{(1)}-H_{\text{ex1}}^{(2)}}{2}+\left(H_{\text{ex2}}^{(1)}-H_{\text{ex2}}^{(2)}\right)\cos\left(\varphi_1+\varphi_2\right)$$

$$A_{34}^3=-\frac{I_1+I_2}{2}+\frac{H_{\text{k1}}}{2}+\frac{H_{\text{k2}}}{2}+\frac{H_{\text{ex1}}^{(1)}+H_{\text{ex1}}^{(2)}}{2}-\left(H_{\text{ex2}}^{(1)}+H_{\text{ex2}}^{(2)}\right)\cos\left(\varphi_1+\varphi_2\right)$$

$$\begin{aligned}A_{41}^3=&\frac{I_1-I_2}{2}-\frac{H_{\text{ex1}}^{(1)}-H_{\text{ex1}}^{(2)}}{2}\cos\left(\varphi_1+\varphi_2\right)+\left(H_{\text{ex2}}^{(1)}-H_{\text{ex2}}^{(2)}\right)\cos^2\left(\varphi_1+\varphi_2\right)\\&-2\left(H_{\text{ex2}}^{(1)}-H_{\text{ex2}}^{(2)}\right)\sin^2\left(\varphi_1+\varphi_2\right)\end{aligned}$$

$$A_{43}^{3}=\frac{I_1+I_2}{2}+\frac{H_{\mathrm{ex1}}^{(1)}+H_{\mathrm{ex1}}^{(2)}}{2}\cos\left(\varphi_1+\varphi_2\right)-\left(H_{\mathrm{ex2}}^{(1)}+H_{\mathrm{ex2}}^{(2)}\right)\cos^2\left(\varphi_1+\varphi_2\right)$$

where

$$\begin{aligned}I_1&=H\sin\varphi_1-H_{\mathrm{ex1}}^{(1)}\cos\left(\varphi_1+\varphi_2\right)+2H_{\mathrm{ex2}}^{(1)}\cos^2\left(\varphi_1+\varphi_2\right)\\I_2&=H\sin\varphi_2-H_{\mathrm{ex1}}^{(2)}\cos\left(\varphi_1+\varphi_2\right)+2H_{\mathrm{ex2}}^{(2)}\cos^2\left(\varphi_1+\varphi_2\right)\end{aligned}$$

And φ_1 and φ_2 are obtained from the following equations:

$$\begin{aligned}-H\cos\varphi_1-H_{\mathrm{ex1}}^{(1)}\sin\left(\varphi_1+\varphi_2\right)+2H_{\mathrm{ex2}}^{(1)}\sin\left(\varphi_1+\varphi_2\right)\cos\left(\varphi_1+\varphi_2\right)=0\\-H\cos\varphi_2-H_{\mathrm{ex1}}^{(2)}\sin\left(\varphi_1+\varphi_2\right)+2H_{\mathrm{ex2}}^{(2)}\sin\left(\varphi_1+\varphi_2\right)\cos\left(\varphi_1+\varphi_2\right)=0\end{aligned}\tag{S-7}$$

For PMA SAF (T-Type), the matrix elements are displayed below:

$$\begin{aligned}
A_{12}^{4}&=\frac{P_1+P_2}{2}-\frac{H_{\mathrm{k1}}}{2}\sin^2\theta_1-\frac{H_{\mathrm{k2}}}{2}\sin^2\theta_2-\frac{H_{\mathrm{ex1}}^{(1)}+H_{\mathrm{ex1}}^{(2)}}{2}\cos\left(\theta_1+\theta_2\right)+\left(H_{\mathrm{ex2}}^{(1)}+H_{\mathrm{ex2}}^{(2)}\right)\cos^2\left(\theta_1+\theta_2\right)\\
&\quad-2\left(H_{\mathrm{ex2}}^{(1)}+H_{\mathrm{ex2}}^{(2)}\right)\sin^2\left(\theta_1+\theta_2\right)\\
A_{14}^{4}&=\frac{P_1-P_2}{2}-\frac{H_{\mathrm{k1}}}{2}\sin^2\theta_1+\frac{H_{\mathrm{k2}}}{2}\sin^2\theta_2+\frac{H_{\mathrm{ex1}}^{(1)}-H_{\mathrm{ex1}}^{(2)}}{2}\cos\left(\theta_1+\theta_2\right)-\left(H_{\mathrm{ex2}}^{(1)}-H_{\mathrm{ex2}}^{(2)}\right)\cos^2\left(\theta_1+\theta_2\right)\\
A_{21}^{4}&=-\frac{P_1+P_2}{2}-\frac{H_{\mathrm{ex1}}^{(1)}+H_{\mathrm{ex1}}^{(2)}}{2}+\left(H_{\mathrm{ex2}}^{(1)}+H_{\mathrm{ex2}}^{(2)}\right)\cos\left(\theta_1+\theta_2\right)\\
A_{23}^{4}&=-\frac{P_1-P_2}{2}+\frac{H_{\mathrm{ex1}}^{(1)}-H_{\mathrm{ex1}}^{(2)}}{2}-\left(H_{\mathrm{ex2}}^{(1)}-H_{\mathrm{ex2}}^{(2)}\right)\cos\left(\theta_1+\theta_2\right)\\
A_{32}^{4}&=\frac{P_1-P_2}{2}-\frac{H_{\mathrm{k1}}}{2}\sin^2\theta_1+\frac{H_{\mathrm{k2}}}{2}\sin^2\theta_2-\frac{H_{\mathrm{ex1}}^{(1)}-H_{\mathrm{ex1}}^{(2)}}{2}\cos\left(\theta_1+\theta_2\right)+\left(H_{\mathrm{ex2}}^{(1)}-H_{\mathrm{ex2}}^{(2)}\right)\cos^2\left(\theta_1+\theta_2\right)\\
&\quad-2\left(H_{\mathrm{ex2}}^{(1)}-H_{\mathrm{ex2}}^{(2)}\right)\sin^2\left(\theta_1+\theta_2\right)\\
A_{34}^{4}&=\frac{P_1+P_2}{2}-\frac{H_{\mathrm{k1}}}{2}\sin^2\theta_1-\frac{H_{\mathrm{k2}}}{2}\sin^2\theta_2+\frac{H_{\mathrm{ex1}}^{(1)}+H_{\mathrm{ex1}}^{(2)}}{2}\cos\left(\theta_1+\theta_2\right)-\left(H_{\mathrm{ex2}}^{(1)}+H_{\mathrm{ex2}}^{(2)}\right)\cos^2\left(\theta_1+\theta_2\right)\\
A_{41}^{4}&=-\frac{P_1-P_2}{2}-\frac{H_{\mathrm{ex1}}^{(1)}-H_{\mathrm{ex1}}^{(2)}}{2}+\left(H_{\mathrm{ex2}}^{(1)}-H_{\mathrm{ex2}}^{(2)}\right)\cos\left(\theta_1+\theta_2\right)\\
A_{43}^{4}&=-\frac{P_1+P_2}{2}+\frac{H_{\mathrm{ex1}}^{(1)}+H_{\mathrm{ex1}}^{(2)}}{2}-\left(H_{\mathrm{ex2}}^{(1)}+H_{\mathrm{ex2}}^{(2)}\right)\cos\left(\theta_1+\theta_2\right)
\end{aligned}$$

where

$$P_1 = \frac{1}{\sin\theta_1}\left[H + H_{\mathrm{ex1}}^{(1)}\sin\theta_2 - 2H_{\mathrm{ex2}}^{(1)}\cos(\theta_1+\theta_2)\sin\theta_2\right]$$
$$P_2 = \frac{1}{\sin\theta_2}\left[H + H_{\mathrm{ex1}}^{(2)}\sin\theta_1 - 2H_{\mathrm{ex2}}^{(2)}\cos(\theta_1+\theta_2)\sin\theta_1\right]$$

And θ_1 and θ_2 in this complete case are obtained from the following equations:

$$\begin{aligned}-H\cos\theta_1 + H_{\mathrm{k1}}\sin\theta_1\cos\theta_1 - H_{\mathrm{ex1}}^{(1)}\sin(\theta_1+\theta_2) + 2H_{\mathrm{ex2}}^{(1)}\sin(\theta_1+\theta_2)\cos(\theta_1+\theta_2) = 0\\ -H\cos\theta_2 + H_{\mathrm{k2}}\sin\theta_2\cos\theta_2 - H_{\mathrm{ex1}}^{(2)}\sin(\theta_1+\theta_2) + 2H_{\mathrm{ex2}}^{(2)}\sin(\theta_1+\theta_2)\cos(\theta_1+\theta_2) = 0\end{aligned} \tag{S-8}$$

We point out that by setting $H_{\mathrm{ex1}}^{(1)} = H_{\mathrm{ex1}}^{(2)} = H_{\mathrm{ex}}$ and $H_{\mathrm{ex2}}^{(1)} = H_{\mathrm{ex2}}^{(2)} = 0$, the matrix elements in the complete case can be transformed into the matrix elements in the simplified case.

Section S4. Resonance features under different field geometries

In this section, we study the influence of different field geometries on the resonance features of SAFs by means of vector network analyzer (VNA) ferromagnetic resonance (FMR) and micromagnetic simulation. We first prepare a reference sample using micro-nano fabrication technique shown in Methods. The structure is: Substrate//Ta (3)/Pt (3)/$Ni_{80}Fe_{20}$ (15)/ Ir (0.45)/ $Ni_{80}Fe_{20}$ (15)/ Ta (3), where the thicknesses in brackets are given in nanometer. In this reference sample, FM1 and FM2 are identical, thus the symmetry of this system is preserved under the joint operations of $C_{2y'}$ and lattice exchange. Figure S3a shows the schematic of VNA FMR measurement setup, where we measure the *S*-parameter to characterize the degree of microwave absorption and thus reflect the resonance information of magnetic moments. An external magnetic field **H** is applied in the sample plane, with an angle of θ_h to the rf magnetic field *h*. For θ_h = 90°, as shown in Fig. S3b, it can be seen that only − (acoustic) mode is excited, and its resonance frequency monotonically increases with the increase of *H*. While for θ_h = 0°, as shown in Fig. S3c, only + (optical) mode is excited. With the increase of *H*, the resonance frequency of + mode shows a slightly increase, and then rapidly decreases to zero. This slightly increase feature also indicates the presence of the biquadratic interlayer exchange in our experimental samples. When θ_h = 45°, both the + mode and − mode can be excited, and the two modes cross without forming a gap, as shown in Fig. S3d. This crossing feature indicates that there is no coupling between the + mode and − mode in this case, which is consistent with the preserved symmetry of the reference sample.

Since our experimental samples S1 to S7, which are shown in the experimental section in the main text, are relatively thin, the VNA FMR method does not have sufficient sensitivity to perform measurements. On the other hand, the microwave field in the ST-FMR measurement is not fixed and difficult to characterize. Therefore, in this section, we perform micromagnetic simulation to further study the resonance features under different field geometries when the system no longer maintains symmetry. We first consider the IP SAF case, as shown in Figs. S4a to S4d. In this case, we consider two examples corresponding to small and large magnetic anisotropic asymmetries. For the example with small magnetic anisotropic asymmetry shown in Figs. S4a and S4b, a gap is clearly shown, indicating the presence of coupling between the + and – modes. Accompanying the appearance of the gap is the excitation of both branches, even when θ_h is equal to 0° or 90°, as shown by the segments in Figs. S4a and S4b. Here, for convenience, we define the two branches as the "major branch" and "minor branch" when specifying θ_h as 0° or 90°. The major (minor) branch represents the branch that can (not) be excited for the specified θ_h when the system is symmetric. For example, in IP SAF, when specifying θ_h as 0°, the branch I (II) is the major (minor) branch when $H < H_0$, while the branch II (I) is the major (minor) branch when $H > H_0$. Although the minor branch can be excited in the presence of coupling, the excitation of the minor branch is still weak when the asymmetry is small, as shown by the segments in the color plots. This feature indicates that the mode hybridization of IP SAF is not sufficient in the case of small magnetic anisotropic asymmetry. For the example with large magnetic anisotropic asymmetry shown in Figs. S4c and S4d, a larger gap is observed. In this case, the excitation of the minor branch is greatly enhanced compared with Figs. S4a and S4b, as shown by the segments in the color plots. This feature indicates that by increasing the degree of magnetic anisotropic asymmetry, the mode hybridization can become more sufficient. We then consider the PMA SAF case, as shown in Figs. S4e to S4h. In this case, similarly, two examples are considered, corresponding to small and large magnetic anisotropic asymmetries, and they have the same degrees of asymmetry $\kappa = (H_{k1}-H_{k2}) / (H_{k1}+H_{k2})$ as the two IP SAF examples, respectively. We find that the PMA SAF case is similar to the IP SAF case in many ways, but there are also differences. For the example with small magnetic anisotropic asymmetry shown in Figs. S4e and S4f, the major branch, is mainly excited in each plot. However, unlike the IP SAF case, the excitation of the minor branch is not weak when H approaches $H_0 = 0$, as shown by the segments in Figs. S4e and S4f. This phenomenon can be explained by the fact that the PMA SAF system has a larger g_1/ω_0 than the IP SAF system when these two systems have the same κ, as

discussed in the main text. With the improvement of magnetic anisotropic asymmetry in PMA SAF, as shown in Figs. S4g and S4h, the mode hybridization becomes more sufficient, and the excitation of minor branch can be equivalent to or even larger than the major branch. We further decrease H_{k1} to −10 kOe, and the magnetization configuration transfers to T-Type, as shown in Figs. S4i and S4j. In this case, though there is no crossing between the + mode and − mode, we note that the major and minor branches are both excited when θ_h = 0° or 90°, indicating that the coupling between the + mode and − mode does occur. Besides, the resonance spectra show interesting features. When H is large, the major branch is mainly excited, while the minor branch is mainly excited when H is small.

Section S5. Quantum description of SAF system

To extract the coupling strength, we quantize our asymmetric SAF system in this section. We consider the simplified case, in which the parameters in FM1 are the same as those in FM2 and the biquadratic exchange interaction is ignored. Moreover, since the interlayer exchange interaction is much larger than the RKKY interaction, we only consider the case with wave number k = 0, which means the magnetic moment in each FM is uniform. $\mathbf{m}_1^{\mathbf{eq}}$ and $\mathbf{m}_2^{\mathbf{eq}}$ are defined as the equilibrium positions of magnetic moments of FM1 and FM2, respectively. The main coordinate systems for PMA SAF (T-Type) and IP SAF are shown in Fig. S5, where we define two local coordinate systems: $\mathbf{e}_{x'(y')(z')}$ for FM1 and $\mathbf{e}_{x''(y'')(z'')}$ for FM2. In PMA SAF (T-Type) case, $\mathbf{e}_{z'} \parallel \mathbf{m}_1^{\mathbf{eq}}$, $\mathbf{e}_{x'} = \mathbf{e}_x$, $\mathbf{e}_{y'} \parallel \mathbf{e}_{z'} \times \mathbf{e}_{x'}$ and $\mathbf{e}_{z''} \parallel \mathbf{m}_2^{\mathbf{eq}}$, $\mathbf{e}_{x''} = \mathbf{e}_x$, $\mathbf{e}_{y''} \parallel \mathbf{e}_{z''} \times \mathbf{e}_{x''}$. And in IP SAF case, $\mathbf{e}_{z'} \parallel \mathbf{m}_1^{\mathbf{eq}}$, $\mathbf{e}_{x'} = \mathbf{e}_z$, $\mathbf{e}_{y'} \parallel \mathbf{e}_{z'} \times \mathbf{e}_{x'}$ and $\mathbf{e}_{z''} \parallel \mathbf{m}_2^{\mathbf{eq}}$, $\mathbf{e}_{x''} = \mathbf{e}_z$, $\mathbf{e}_{y''} \parallel \mathbf{e}_{z''} \times \mathbf{e}_{x''}$. The r axis is defined to be parallel to $\mathbf{m}_1^{\mathbf{eq}} + \mathbf{m}_2^{\mathbf{eq}}$. In IP SAF case, the r axis and y axis coincide. The magnetic field $\mathbf{H}$ is applied in the y axis and $\theta_{1(2)}$, $\varphi_{1(2)}$ follow the definitions in the main text. Considering the system with unit volume, the classical Hamiltonian of the system can be written as

$$\mathcal{H} = \mathcal{H}_{\text{Zeeman}} + \mathcal{H}_{\text{Ani}} + \mathcal{H}_{\text{Exchange}} \tag{S-9}$$

with

$$\begin{aligned}
\mathcal{H}_{\text{Zeeman}} &= -\mu_0 \mathbf{H} \cdot \mathbf{M}_{\mathbf{S1}} - \mu_0 \mathbf{H} \cdot \mathbf{M}_{\mathbf{S2}} \\
\mathcal{H}_{\text{Ani}} &= -\mu_0 \frac{H_{\text{k1}}}{2M_{\text{S}}} \left(\mathbf{M}_{\mathbf{S1}} \cdot \mathbf{e}_{\mathbf{z}}\right)^2 - \mu_0 \frac{H_{\text{k2}}}{2M_{\text{S}}} \left(\mathbf{M}_{\mathbf{S2}} \cdot \mathbf{e}_{\mathbf{z}}\right)^2 \\
\mathcal{H}_{\text{Exchange}} &= -\mu_0 \frac{H_{\text{ex}}}{M_{\text{S}}} \left(\mathbf{M}_{\mathbf{S1}} \cdot \mathbf{M}_{\mathbf{S2}}\right)
\end{aligned}$$

where $\mathbf{M_{S1}} = M_{S1,x'}\mathbf{e_{x'}} + M_{S1,y'}\mathbf{e_{y'}} + M_{S1,z'}\mathbf{e_{z'}}$, $\mathbf{M_{S2}} = M_{S2,x''}\mathbf{e_{x''}} + M_{S2,y''}\mathbf{e_{y''}} + M_{S2,z''}\mathbf{e_{z''}}$ correspond to magnetization vectors of FM1 and FM2, respectively. And $M_S = |\mathbf{M_{S1}}| = |\mathbf{M_{S2}}|$ corresponds to the saturation magnetization. We define an operator C_{2r} which rotates vectors 180° about the r axis. And we define the following magnetization vector for convenience: $\mathbf{M'_{S2}} = C_{2r}\mathbf{M_{S2}}$, where $\mathbf{M'_{S2}} = M_{S2,x'}\mathbf{e_{x'}} + M_{S2,y'}\mathbf{e_{y'}} + M_{S2,z'}\mathbf{e_{z'}}$. $M_{S2,x'}$, $M_{S2,y'}$ and $M_{S2,z'}$ satisfy the following relations to $\mathbf{M_{S2}}$: $M_{S2,x'} = -M_{S2,x''}$, $M_{S2,y'} = -M_{S2,y''}$ and $M_{S2,z'} = M_{S2,z''}$. We then define two combined magnetic moment components: $M^{+}_{i'} = M_{S1,i'} + M_{S2,i'}$ and $M^{-}_{i'} = M_{S1,i'} - M_{S2,i'}$ ($i = x, y, z$). We note that when $i = x$ and y, $M^{+}_{i'}$ and $M^{-}_{i'}$ correspond to the macrospin joint precession vectors $\delta\mathbf{m}_+ = \delta\mathbf{m_1} + C_{2y'}\delta\mathbf{m_2}$ and $\delta\mathbf{m}_- = \delta\mathbf{m_1} - C_{2y'}\delta\mathbf{m_2}$ shown in Methods, respectively. Therefore, $M^{+}_{i'}$ and $M^{-}_{i'}$ relate to pure + (optical) mode and − (acoustic) mode, respectively. Then, we rewrite Eq. S-9 by substituting $M^{+}_{i'}$ and $M^{-}_{i'}$ into the formula. For IP SAF:

$$\mathcal{H}_{\text{Zeeman}} = -\mu_0 H\left(\cos\varphi M^{+}_{y'} + \sin\varphi M^{+}_{z'}\right)$$

$$\mathcal{H}_{\text{Ani}} = -\frac{\mu_0}{8M_S}\left[\left(H_{k1}+H_{k2}\right)\left(M^{+2}_{x'}+M^{-2}_{x'}\right)+\left(H_{k1}-H_{k2}\right)\left(M^{+}_{x'}M^{-}_{x'}+M^{-}_{x'}M^{+}_{x'}\right)\right]$$

$$\mathcal{H}_{\text{Exchange}} = -\mu_0\frac{H_{ex}}{4M_S}\Big[-M^{+2}_{x'}+M^{-2}_{x'}+\cos 2\varphi\left(M^{+2}_{y'}-M^{-2}_{y'}-M^{+2}_{z'}+M^{-2}_{z'}\right)$$
$$+2\sin 2\varphi\left(M^{+}_{y'}M^{+}_{z'}-M^{-}_{y'}M^{-}_{z'}\right)\Big]$$

where $\varphi = \varphi_1 = \varphi_2 = \arcsin(H/-2H_{ex})$ and we only consider the case with $H < -2H_{ex}$ for IP SAF here. For PMA SAF (T-Type), Equation S-9 can be rewritten as

$$\mathcal{H}_{\text{Zeeman}} = -\frac{\mu_0 H}{2}\Big[\left(\cos\theta_1+\cos\theta_2\right)M^{+}_{y'}+\left(\cos\theta_1-\cos\theta_2\right)M^{-}_{y'}$$
$$+\left(\sin\theta_1+\sin\theta_2\right)M^{+}_{z'}+\left(\sin\theta_1-\sin\theta_2\right)M^{-}_{z'}\Big]$$

$$\mathcal{H}_{\text{Ani}} = -\frac{\mu_0}{8M_S}\Big[\left(H_{k1}\sin^2\theta_1+H_{k2}\sin^2\theta_2\right)\left(M^{+2}_{y'}+M^{-2}_{y'}\right)+\left(H_{k1}\cos^2\theta_1+H_{k2}\cos^2\theta_2\right)\left(M^{+2}_{z'}+M^{-2}_{z'}\right)$$
$$-\left(H_{k1}\sin\theta_1\cos\theta_1+H_{k2}\sin\theta_2\cos\theta_2\right)\left(M^{+}_{y'}M^{+}_{z'}+M^{+}_{z'}M^{+}_{y'}+M^{-}_{y'}M^{-}_{z'}+M^{-}_{z'}M^{-}_{y'}\right)$$
$$+\left(H_{k1}\sin^2\theta_1-H_{k2}\sin^2\theta_2\right)\left(M^{+}_{y'}M^{-}_{y'}+M^{-}_{y'}M^{+}_{y'}\right)+\left(H_{k1}\cos^2\theta_1-H_{k2}\cos^2\theta_2\right)\left(M^{+}_{z'}M^{-}_{z'}+M^{-}_{z'}M^{+}_{z'}\right)$$
$$-\left(H_{k1}\sin\theta_1\cos\theta_1-H_{k2}\sin\theta_2\cos\theta_2\right)\left(M^{+}_{y'}M^{-}_{z'}+M^{-}_{z'}M^{+}_{y'}+M^{-}_{y'}M^{+}_{z'}+M^{+}_{z'}M^{-}_{y'}\right)\Big]$$

$$\mathcal{H}_{\text{Exchange}} = -\mu_0\frac{H_{ex}}{4M_S}\Big[-M^{+2}_{x'}+M^{-2}_{x'}+\cos\left(\theta_1+\theta_2\right)\left(M^{+2}_{y'}-M^{-2}_{y'}-M^{+2}_{z'}+M^{-2}_{z'}\right)$$
$$+2\sin\left(\theta_1+\theta_2\right)\left(M^{+}_{y'}M^{+}_{z'}-M^{-}_{y'}M^{-}_{z'}\right)\Big]$$

where θ_1 and θ_2 are derived from Eq. S-6. The above Hamiltonian can be divided into two parts, one of which can be expressed as the sum of pure + mode and − mode, and is defined as a pure + mode and − mode Hamiltonian, while the other part is a coupled Hamiltonian that cannot be expressed as the sum of the two pure modes. Note that $M_{z'}^{+}$ and $M_{z'}^{-}$ are not independent, they are related to $M_{i'}^{+}$ and $M_{i'}^{-}$ $(i = x, y)$:

$$\begin{aligned} M_{z'}^{+} &= 2M_S - \frac{M_{y'}^{+2} + M_{y'}^{-2}}{4M_S} - \frac{M_{x'}^{+2} + M_{x'}^{-2}}{4M_S} \\ M_{z'}^{-} &= -\frac{M_{y'}^{+}M_{y'}^{-} + M_{y'}^{-}M_{y'}^{+}}{4M_S} - \frac{M_{x'}^{+}M_{x'}^{-} + M_{x'}^{-}M_{x'}^{+}}{4M_S} \end{aligned} \tag{S-10}$$

Therefore

$$\mathcal{H} = \mathcal{H}_{\text{Pure}} + \mathcal{H}_{\text{Couple}} \tag{S-11}$$

For IP SAF:

$$\begin{aligned} \mathcal{H}_{\text{Pure}} = &-\mu_0 H\left(\cos\varphi M_{y'}^{+} + \sin\varphi M_{z'}^{+}\right) - \frac{\mu_0}{8M_S}\left(H_{k1} + H_{k2}\right)\left(M_{x'}^{+2} + M_{x'}^{-2}\right) \\ &-\mu_0 \frac{H_{ex}}{4M_S}\left[-M_{x'}^{+2} + M_{x'}^{-2} + \cos 2\varphi\left(M_{y'}^{+2} - M_{y'}^{-2} - M_{z'}^{+2}\right) + 4\sin 2\varphi M_S M_{y'}^{+}\right] \end{aligned}$$

$$\mathcal{H}_{\text{Couple}} = -\frac{\mu_0}{8M_S}\left(H_{k1} - H_{k2}\right)\left(M_{x'}^{+}M_{x'}^{-} + M_{x'}^{-}M_{x'}^{+}\right)$$

And for PMA SAF (T-Type):

$$\begin{aligned} \mathcal{H}_{\text{Pure}} = &-\frac{\mu_0 H}{2}\left[\left(\cos\theta_1 + \cos\theta_2\right)M_{y'}^{+} + \left(\cos\theta_1 - \cos\theta_2\right)M_{y'}^{-} + \left(\sin\theta_1 + \sin\theta_2\right)M_{z'}^{+}\right] \\ &-\frac{\mu_0}{8M_S}\Big[\left(H_{k1}\sin^2\theta_1 + H_{k2}\sin^2\theta_2\right)\left(M_{y'}^{+2} + M_{y'}^{-2}\right) + \left(H_{k1}\cos^2\theta_1 + H_{k2}\cos^2\theta_2\right)M_{z'}^{+2} \\ &-4\left(H_{k1}\sin\theta_1\cos\theta_1 + H_{k2}\sin\theta_2\cos\theta_2\right)M_S M_{y'}^{+} - 4\left(H_{k1}\sin\theta_1\cos\theta_1 - H_{k2}\sin\theta_2\cos\theta_2\right)M_S M_{y'}^{-}\Big] \\ &-\mu_0\frac{H_{ex}}{4M_S}\left[-M_{x'}^{+2} + M_{x'}^{-2} + \cos\left(\theta_1 + \theta_2\right)\left(M_{y'}^{+2} - M_{y'}^{-2} - M_{z'}^{+2}\right) + 4\sin\left(\theta_1 + \theta_2\right)M_S M_{y'}^{+}\right] \end{aligned}$$

$$\mathcal{H}_{\text{Couple}} = -\frac{\mu_0 H}{2}\left(\sin\theta_1 - \sin\theta_2\right)M_{\text{z'}}^{-}$$
$$-\frac{\mu_0}{8M_{\text{S}}}\left[\left(H_{\text{k1}}\sin^2\theta_1 - H_{\text{k2}}\sin^2\theta_2\right)\left(M_{\text{y'}}^{+}M_{\text{y'}}^{-} + M_{\text{y'}}^{-}M_{\text{y'}}^{+}\right) + 4\left(H_{\text{k1}}\cos^2\theta_1 - H_{\text{k2}}\cos^2\theta_2\right)M_{\text{S}}M_{\text{z'}}^{-}\right]$$

where we ignore small quantities above the second order.

We now quantize the Hamiltonian Eq. S-11. The Holstein-Primakoff (HP) transformation[1] is given by

$$\begin{aligned}
\hat{M}_{\text{S1,z'}} &= M_{\text{S}} - \hbar\gamma_0 \hat{a}_1^{\dagger}\hat{a}_1 \\
\hat{M}_{\text{S2,z'}} &= M_{\text{S}} - \hbar\gamma_0 \hat{a}_2^{\dagger}\hat{a}_2 \\
\hat{M}_{\text{S1,+}} &= \hat{M}_{\text{S1,x'}} - \mathrm{i}\,\hat{M}_{\text{S1,y'}} = \sqrt{2\gamma_0\hbar M_{\text{S}}}\,\hat{a}_1 \\
\hat{M}_{\text{S2,+}} &= \hat{M}_{\text{S2,x'}} - \mathrm{i}\,\hat{M}_{\text{S2,y'}} = \sqrt{2\gamma_0\hbar M_{\text{S}}}\,\hat{a}_2 \\
\hat{M}_{\text{S1,-}} &= \hat{M}_{\text{S1,x'}} + \mathrm{i}\,\hat{M}_{\text{S1,y'}} = \sqrt{2\gamma_0\hbar M_{\text{S}}}\,\hat{a}_1^{\dagger} \\
\hat{M}_{\text{S2,-}} &= \hat{M}_{\text{S2,x'}} + \mathrm{i}\,\hat{M}_{\text{S2,y'}} = \sqrt{2\gamma_0\hbar M_{\text{S}}}\,\hat{a}_2^{\dagger}
\end{aligned} \tag{S-12}$$

Thus

$$\begin{aligned}
\hat{M}_{\text{z'}}^{+} &= \hat{M}_{\text{S1,z'}} + \hat{M}_{\text{S2,z'}} = 2M_{\text{S}} - \hbar\gamma_0\hat{a}_1^{\dagger}\hat{a}_1 - \hbar\gamma_0\hat{a}_2^{\dagger}\hat{a}_2 \\
\hat{M}_{\text{z'}}^{-} &= \hat{M}_{\text{S1,z'}} - \hat{M}_{\text{S2,z'}} = \hbar\gamma_0\hat{a}_2^{\dagger}\hat{a}_2 - \hbar\gamma_0\hat{a}_1^{\dagger}\hat{a}_1 \\
\hat{M}_{\text{x'}}^{+} &= \hat{M}_{\text{S1,x'}} + \hat{M}_{\text{S2,x'}} = \frac{1}{2}\sqrt{2\gamma_0\hbar M_{\text{S}}}\left(\hat{a}_1 + \hat{a}_1^{\dagger} + \hat{a}_2 + \hat{a}_2^{\dagger}\right) \\
\hat{M}_{\text{x'}}^{-} &= \hat{M}_{\text{S1,x'}} - \hat{M}_{\text{S2,x'}} = \frac{1}{2}\sqrt{2\gamma_0\hbar M_{\text{S}}}\left(\hat{a}_1 + \hat{a}_1^{\dagger} - \hat{a}_2 - \hat{a}_2^{\dagger}\right) \\
\hat{M}_{\text{y'}}^{+} &= \hat{M}_{\text{S1,y'}} + \hat{M}_{\text{S2,y'}} = \frac{\mathrm{i}}{2}\sqrt{2\gamma_0\hbar M_{\text{S}}}\left(\hat{a}_1 - \hat{a}_1^{\dagger} + \hat{a}_2 - \hat{a}_2^{\dagger}\right) \\
\hat{M}_{\text{y'}}^{-} &= \hat{M}_{\text{S1,y'}} - \hat{M}_{\text{S2,y'}} = \frac{\mathrm{i}}{2}\sqrt{2\gamma_0\hbar M_{\text{S}}}\left(\hat{a}_1 - \hat{a}_1^{\dagger} - \hat{a}_2 + \hat{a}_2^{\dagger}\right)
\end{aligned} \tag{S-13}$$

where $\gamma_0 = \gamma/\mu_0$. The quantized Hamiltonian is obtained by substituting the quantized physical quantities into the Eq. S-11

$$\hat{\mathcal{H}} = \hat{\mathcal{H}}_{\text{Pure}} + \hat{\mathcal{H}}_{\text{Couple}} \tag{S-14}$$

For IP SAF:

$$
\begin{aligned}
\hat{\mathcal{H}}_{\text{Pure}} = \hat{\mathcal{H}}_0 + \Bigg\{ & \frac{1}{2}\left[-\frac{1}{4}\hbar\mu_0\gamma_0\left(H_{\text{k}1}+H_{\text{k}2}\right) - \hbar\mu_0\gamma_0 H_{\text{ex}} \right]\left(\hat{a}_1^\dagger\hat{a}_1 + \hat{a}_2^\dagger\hat{a}_2\right) \\
& -\frac{1}{8}\hbar\mu_0\gamma_0\left(H_{\text{k}1}+H_{\text{k}2}\right)\left(\hat{a}_1^2+\hat{a}_2^2\right) + \hbar\mu_0\gamma_0\left(H_{\text{ex}} - \frac{H^2}{4H_{\text{ex}}}\right)\hat{a}_1\hat{a}_2 + \frac{1}{4}\hbar\mu_0\gamma_0\frac{H^2}{H_{\text{ex}}}\hat{a}_1^\dagger\hat{a}_2 \\
& + \left[\frac{\mathrm{i}}{4}\mu_0 H\sqrt{2\gamma_0\hbar M_{\text{S}}}\frac{\sqrt{4H_{\text{ex}}^2-H^2}}{H_{\text{ex}}} - \frac{\mathrm{i}}{4}\mu_0\sqrt{2\gamma_0\hbar M_{\text{S}}}\frac{H\sqrt{4H_{\text{ex}}^2-H^2}}{H_{\text{ex}}} \right]\hat{a}_1 \\
& + \left[\frac{\mathrm{i}}{4}\mu_0 H\sqrt{2\gamma_0\hbar M_{\text{S}}}\frac{\sqrt{4H_{\text{ex}}^2-H^2}}{H_{\text{ex}}} - \frac{\mathrm{i}}{4}\mu_0\sqrt{2\gamma_0\hbar M_{\text{S}}}\frac{H\sqrt{4H_{\text{ex}}^2-H^2}}{H_{\text{ex}}} \right]\hat{a}_2 \\
& + \text{h.c.} \Bigg\} \\
= \hat{\mathcal{H}}_0 + & \left[\frac{1}{2}A_{\text{pure}}^1\left(\hat{a}_1^\dagger\hat{a}_1 + \hat{a}_2^\dagger\hat{a}_2\right) + B_{\text{pure}}^1\left(\hat{a}_1^2+\hat{a}_2^2\right) + C_{\text{pure}}^1\hat{a}_1\hat{a}_2 + D_{\text{pure}}^1\hat{a}_1^\dagger\hat{a}_2 + e_{\text{pure}}^1\hat{a}_1 + f_{\text{pure}}^1\hat{a}_2 + \text{h.c.} \right]
\end{aligned}
$$

$$
\hat{\mathcal{H}}_{\text{Couple}} = -\frac{1}{8}\hbar\mu_0\gamma_0\left(H_{\text{k}1}-H_{\text{k}2}\right)\left(\hat{a}_1^2 - \hat{a}_2^2 + \hat{a}_1^\dagger\hat{a}_1 - \hat{a}_2^\dagger\hat{a}_2\right) + \text{h.c.}
$$

And for PMA SAF (T-Type):

$$
\begin{aligned}
\hat{\mathcal{H}}_{\text{Pure}} = \hat{\mathcal{H}}_0 + \Bigg\{ & \frac{1}{2}\Bigg[\frac{1}{2}\hbar\mu_0\gamma_0 H\left(\sin\theta_1+\sin\theta_2\right) - \frac{1}{4}\hbar\mu_0\gamma_0\left(H_{\text{k}1}\sin^2\theta_1 + H_{\text{k}2}\sin^2\theta_2\right) \\
& + \frac{1}{2}\hbar\mu_0\gamma_0\left(H_{\text{k}1}\cos^2\theta_1 + H_{\text{k}2}\cos^2\theta_2\right) - \hbar\mu_0\gamma_0 H_{\text{ex}}\cos\left(\theta_1+\theta_2\right)\Bigg]\left(\hat{a}_1^\dagger\hat{a}_1 + \hat{a}_2^\dagger\hat{a}_2\right) \\
& + \frac{1}{8}\hbar\mu_0\gamma_0\left(H_{\text{k}1}\sin^2\theta_1 + H_{\text{k}2}\sin^2\theta_2\right)\left(\hat{a}_1^2+\hat{a}_2^2\right) \\
& + \frac{1}{2}\hbar\mu_0\gamma_0 H_{\text{ex}}\left[1+\cos\left(\theta_1+\theta_2\right)\right]\hat{a}_1\hat{a}_2 + \frac{1}{2}\hbar\mu_0\gamma_0 H_{\text{ex}}\left[1-\cos\left(\theta_1+\theta_2\right)\right]\hat{a}_1^\dagger\hat{a}_2 \\
& + \left[-\frac{\mathrm{i}}{2}\mu_0 H\sqrt{2\gamma_0\hbar M_{\text{S}}}\cos\theta_1 + \frac{\mathrm{i}}{2}\mu_0 H_{\text{k}1}\sqrt{2\gamma_0\hbar M_{\text{S}}}\sin\theta_1\cos\theta_1 - \frac{\mathrm{i}}{2}\mu_0 H_{\text{ex}}\sqrt{2\gamma_0\hbar M_{\text{S}}}\sin\left(\theta_1+\theta_2\right) \right]\hat{a}_1 \\
& + \left[-\frac{\mathrm{i}}{2}\mu_0 H\sqrt{2\gamma_0\hbar M_{\text{S}}}\cos\theta_2 + \frac{\mathrm{i}}{2}\mu_0 H_{\text{k}2}\sqrt{2\gamma_0\hbar M_{\text{S}}}\sin\theta_1\cos\theta_1 - \frac{\mathrm{i}}{2}\mu_0 H_{\text{ex}}\sqrt{2\gamma_0\hbar M_{\text{S}}}\sin\left(\theta_1+\theta_2\right) \right]\hat{a}_2 \\
& + \text{h.c.} \Bigg\} \\
= \hat{\mathcal{H}}_0 + & \left[\frac{1}{2}A_{\text{pure}}^2\left(\hat{a}_1^\dagger\hat{a}_1 + \hat{a}_2^\dagger\hat{a}_2\right) + B_{\text{pure}}^2\left(\hat{a}_1^2+\hat{a}_2^2\right) + C_{\text{pure}}^2\hat{a}_1\hat{a}_2 + D_{\text{pure}}^2\hat{a}_1^\dagger\hat{a}_2 + e_{\text{pure}}^2\hat{a}_1 + f_{\text{pure}}^2\hat{a}_2 + \text{h.c.} \right]
\end{aligned}
$$

$$\hat{\mathcal{H}}_{\text{Couple}} = \frac{1}{2}\Bigg[\frac{1}{2}\hbar\mu_0\gamma_0 H\left(\sin\theta_1 - \sin\theta_2\right) - \frac{1}{4}\hbar\mu_0\gamma_0\left(H_{\text{k1}}\sin^2\theta_1 - H_{\text{k2}}\sin^2\theta_2\right)$$
$$+\frac{1}{2}\hbar\mu_0\gamma_0\left(H_{\text{k1}}\cos^2\theta_1 - H_{\text{k2}}\cos^2\theta_2\right)\Bigg]\hat{a}_1^\dagger\hat{a}_1$$
$$+\frac{1}{2}\Bigg[\frac{1}{2}\hbar\mu_0\gamma_0 H\left(\sin\theta_2 - \sin\theta_1\right) + \frac{1}{4}\hbar\mu_0\gamma_0\left(H_{\text{k1}}\sin^2\theta_1 - H_{\text{k2}}\sin^2\theta_2\right)$$
$$-\frac{1}{2}\hbar\mu_0\gamma_0\left(H_{\text{k1}}\cos^2\theta_1 - H_{\text{k2}}\cos^2\theta_2\right)\Bigg]\hat{a}_2^\dagger\hat{a}_2$$
$$+\frac{1}{8}\hbar\mu_0\gamma_0\left(H_{\text{k1}}\sin^2\theta_1 - H_{\text{k2}}\sin^2\theta_2\right)\left(\hat{a}_1^2 - \hat{a}_2^2\right) + \text{h.c.}$$

where $\hat{\mathcal{H}}_0$ corresponds to the constant term. Then, we perform Bogoliubov transformation to diagonalize the pure Hamiltonian $\hat{\mathcal{H}}_{\text{Pure}}$. We first define the following pure + mode and − mode Boson operators

$$\begin{aligned}\hat{a}_+ &= w_+^{\text{k}}\hat{a}_1 + x_+^{\text{k}}\hat{a}_2 + y_+^{\text{k}}\hat{a}_1^\dagger + z_+^{\text{k}}\hat{a}_2^\dagger + \lambda_+^{\text{k}}\\ \hat{a}_- &= w_-^{\text{k}}\hat{a}_1 + x_-^{\text{k}}\hat{a}_2 + y_-^{\text{k}}\hat{a}_1^\dagger + z_-^{\text{k}}\hat{a}_2^\dagger + \lambda_-^{\text{k}}\end{aligned} \tag{S-15}$$

where $k \in \{1,2\}$, $k = 1$ corresponds to IP SAF case, and $k = 2$ corresponds to PMA SAF (T-Type) case. Boson commutation rules require

$$w_{\text{i}}^{\text{k}} w_{\text{j}}^{\text{k}*} + x_{\text{i}}^{\text{k}} x_{\text{j}}^{\text{k}*} - y_{\text{i}}^{\text{k}} y_{\text{j}}^{\text{k}*} - z_{\text{i}}^{\text{k}} z_{\text{j}}^{\text{k}*} = \delta_{\text{ij}},\ i(j) \in \{+,-\}$$

The corresponding inverse transformation is

$$\begin{aligned}\hat{a}_1 &= w_+^{\text{k}*}\left(\hat{a}_+ - \lambda_+^{\text{k}}\right) + w_-^{\text{k}*}\left(\hat{a}_- - \lambda_-^{\text{k}}\right) - y_+^{\text{k}}\left(\hat{a}_+^\dagger - \lambda_+^{\text{k}*}\right) - y_-^{\text{k}}\left(\hat{a}_-^\dagger - \lambda_-^{\text{k}*}\right)\\ \hat{a}_2 &= x_+^{\text{k}*}\left(\hat{a}_+ - \lambda_+^{\text{k}}\right) + x_-^{\text{k}*}\left(\hat{a}_- - \lambda_-^{\text{k}}\right) - z_+^{\text{k}}\left(\hat{a}_+^\dagger - \lambda_+^{\text{k}*}\right) - z_-^{\text{k}}\left(\hat{a}_-^\dagger - \lambda_-^{\text{k}*}\right)\end{aligned} \tag{S-16}$$

We note that by selecting appropriate parameters λ_+^{k} and λ_-^{k}, linear term in Hamiltonian Eq. S-14 can be eliminated. Besides, the constant term in Hamiltonian does not affect dynamic responses. Therefore, we only consider the quadratic terms below. The Heisenberg equations of motion are

$$\frac{\mathrm{d}\hat{a}_{+(-)}}{\mathrm{d}t} = -\mathrm{i}\,\omega_{+(-)}\hat{a}_{+(-)} = \frac{1}{\mathrm{i}\hbar}\left[\hat{a}_{+(-)}, \hat{\mathcal{H}}_{\text{Pure}}\right] \tag{S-17}$$

which correspond to a fourth order matrix equation

$$\hbar\omega_{+(-)}\begin{bmatrix} w_{+(-)}^{\mathrm{k}} \\ x_{+(-)}^{\mathrm{k}} \\ y_{+(-)}^{\mathrm{k}} \\ z_{+(-)}^{\mathrm{k}} \end{bmatrix} = \begin{bmatrix} A_{\mathrm{pure}}^{\mathrm{k}} & D_{\mathrm{pure}}^{\mathrm{k}} & -2B_{\mathrm{pure}}^{\mathrm{k}} & -C_{\mathrm{pure}}^{\mathrm{k}} \\ D_{\mathrm{pure}}^{\mathrm{k}} & A_{\mathrm{pure}}^{\mathrm{k}} & -C_{\mathrm{pure}}^{\mathrm{k}} & -2B_{\mathrm{pure}}^{\mathrm{k}} \\ 2B_{\mathrm{pure}}^{\mathrm{k}} & C_{\mathrm{pure}}^{\mathrm{k}} & -A_{\mathrm{pure}}^{\mathrm{k}} & -D_{\mathrm{pure}}^{\mathrm{k}} \\ C_{\mathrm{pure}}^{\mathrm{k}} & 2B_{\mathrm{pure}}^{\mathrm{k}} & -D_{\mathrm{pure}}^{\mathrm{k}} & -A_{\mathrm{pure}}^{\mathrm{k}} \end{bmatrix}\begin{bmatrix} w_{+(-)}^{\mathrm{k}} \\ x_{+(-)}^{\mathrm{k}} \\ y_{+(-)}^{\mathrm{k}} \\ z_{+(-)}^{\mathrm{k}} \end{bmatrix}$$

The eigenfrequencies and eigenvectors can be obtained by solving this equation. We note that the solutions ω_+ and ω_- can be expressed in the forms of the parameters in macrospin approach, which are displayed in Eq. 6 in Methods. We point out that $\hat{a}_{+(-)}$ corresponds to a linear superposition of $\hat{M}_{\mathrm{x'}}^{+(-)}$ and $\hat{M}_{\mathrm{y'}}^{+(-)}$, which prove that its corresponding quasiparticle is pure + (–) mode magnon. And the diagonalized pure Hamiltonian $\hat{\mathcal{H}}_{\mathrm{Pure}}$ can now be written as the sum of two decoupled harmonic oscillators:

$$\hat{\mathcal{H}}_{\mathrm{Pure}} = \hbar\omega_+\left(\hat{a}_+^\dagger\hat{a}_+ + \frac{1}{2}\right) + \hbar\omega_-\left(\hat{a}_-^\dagger\hat{a}_- + \frac{1}{2}\right) \tag{S-18}$$

A complete set of base vectors for this Hamiltonian is

$$\left|n_+, m_-\right\rangle_{\mathrm{d}} = \left|n_+\right\rangle \otimes \left|m_-\right\rangle,\ n(m) \in \{0,1,2\cdots\}$$

where n_+ (m_-) represents the number of the + (–) mode magnon. Now we substitute the inverse transformation Eq. S-16 into $\hat{\mathcal{H}}_{\mathrm{Couple}}$. By selecting appropriate phase, we obtain

$$\hat{\mathcal{H}}_{\mathrm{Couple}} = \mathrm{i}\hbar g_1\left(\hat{a}_+\hat{a}_-^\dagger - \hat{a}_+^\dagger\hat{a}_-\right) + \mathrm{i}\hbar g_2\left(\hat{a}_+^\dagger\hat{a}_-^\dagger - \hat{a}_+\hat{a}_-\right) \tag{S-19}$$

where g_1 and g_2 can also be expressed in the forms of the parameters in macrospin approach, which are displayed in Eq. 6 in Methods. Therefore, the Hamiltonian $\hat{\mathcal{H}}$ corresponds to the generalized Hopfield model, as shown in Eq. 1 in the main text.

In the above derivation, it can be found that for PMA SAF (T-Type) system, the coupled Hamiltonian $\mathcal{H}_{\mathrm{Couple}}$ in Eq. S-11 does not include the exchange interaction energy $\mathcal{H}_{\mathrm{Exchange}}$, and for IP SAF system, $\mathcal{H}_{\mathrm{Couple}}$ even includes only the anisotropy energy $\mathcal{H}_{\mathrm{Ani}}$. However, the derived g_1 and g_2 in both systems are not only influenced by the magnetic anisotropy, but also by H and H_{ex}, as shown in the main text and the following Section S6. We point out two reasons for this

result. The minor reason is that $\mathcal{H}_{\text{Couple}}$ for PMA SAF (T-Type) includes θ_1 and θ_2, while $\theta_{1(2)}$ is solved numerically from Eq. S-6, which is a function of H_{ex} and other parameters. Thus, this reason partly accounts for the result that the coupling strengths are related to H_{ex} in PMA SAF (T-Type) system. The major reason is that in Heisenberg equations of motion Eq. S-17, the matrix elements $A^{\text{k}}_{\text{pure}}$, $B^{\text{k}}_{\text{pure}}$, $C^{\text{k}}_{\text{pure}}$, $D^{\text{k}}_{\text{pure}}$ are not constants, but functions of the parameters of the system and the external field. Thus, the solved Bogoliubov transformation coefficients $w^{\text{k}}_{+(-)}$, $x^{\text{k}}_{+(-)}$, $y^{\text{k}}_{+(-)}$, $z^{\text{k}}_{+(-)}$ are also not constants, but functions of the parameters of the system and the external field. Therefore, when applying the inverse transformation Eq. S-16 to $\hat{\mathcal{H}}_{\text{Couple}}$, H and H_{ex} are introduced into the expression of $g_{1(2)}$. This reason also shows the flexibility of magnon. For the quanta of + (−) mode, their properties are not invariable, but can be tuned by modifying the intrinsic properties of the systems and external conditions. In this section, although the derivation is based on the simplified case, we point out that the obtained results are also applicable to the complete case.

Section S6. Effect of RKKY interaction strength on coupling properties

In Fig. S6, we draw the color plots of calculated g_1/ω_0 and g_2/ω_0 as functions of H_{k1} and H_{k2} with different H_{ex} of −0.5, −1 and −3 kOe, respectively. As can be seen, the main properties derived from Fig. 2 in the main text remain unchanged when H_{ex} changes, such as zero g_2/ω_0 in PMA SAF region and $g_1/\omega_0 = 1$ at the PMA SAF-T-Type boundary. The difference is that with the decrease of the absolute value of H_{ex}, the PMA SAF and IP SAF regions shrink while the T-Type regions expand, which can be deduced from Section S2. And the maximum value of g_2/ω_0 increases from below 0.6 to about 0.68 when $|H_{\text{ex}}|$ decreases from 3 kOe to 0.5 kOe.

In order to display the relationship between the coupling strengths and H_{ex} more intuitively, we further display the color plots of calculated $g_{1(2)}/\omega_0$ as a function of H_{ex} and H_{k1}, as shown in Fig. S7. It can be seen from the color plots that $g_{1(2)}/\omega_0$ is related to H_{ex}. However, in most cases, $g_{1(2)}/\omega_0$ does not change as much with H_{ex} as it does with $H_{\text{k1(2)}}$. In PMA SAF region, g_1/ω_0 slightly increases with the decrease of $|H_{\text{ex}}|$, and g_2/ω_0 remains zero. Similarly, in IP SAF region, $g_{1(2)}/\omega_0$ slightly increases with the decrease of $|H_{\text{ex}}|$. For these two cases, H_{ex} plays a minor role in tuning $g_{1(2)}/\omega_0$. And $g_{1(2)}/\omega_0$ as functions of H_{ex} can be described by Eq. 8 and Eq. 9, which have simple forms. However, in T-Type regions, the relationship $g_{1(2)}/\omega_0$-H_{ex} becomes a bit more complicated. This is because the equilibrium positions of $\mathbf{m}_1$ and $\mathbf{m}_2$ are directly modified by H_{ex}

for T-Type when $H = H_0 = 0$. When $H_{k2} = 5$ kOe and $|H_{k1}|$ is large, $g_{1(2)}/\omega_0$ also increases with the decrease of $|H_{ex}|$, but the increase is more rapid than that of PMA SAF and IP SAF, as shown in Figs. S7a and S7b. However, when H_{k1} approaches the boundary between PMA SAF and T-Type, g_1/ω_0 can decrease with the decrease of $|H_{ex}|$. When $H_{k2} = -5$ kOe, one striking feature is that g_2/ω_0 can be largely enhanced near the boundary of IP SAF and T-Type when H_{ex} is small. Thus, we can obtain an extremely large $g_2/\omega_0 \sim 0.9$ in T-Type configuration by modifying H_{ex} and $H_{k1(2)}$.

Section S7. Derivation of the quantum fluctuation and average magnon number in SAF system and some typical examples

The Hamiltonian Eq. 1 can be diagonalized by the Hopfield-Bogoliubov transformation:

$$\hat{\mathcal{H}} = \hbar\omega_{\mathrm{I}}\left(\hat{p}_{\mathrm{I}}^{\dagger}\hat{p}_{\mathrm{I}} + \frac{1}{2}\right) + \hbar\omega_{\mathrm{II}}\left(\hat{p}_{\mathrm{II}}^{\dagger}\hat{p}_{\mathrm{II}} + \frac{1}{2}\right) \tag{S-20}$$

where $\hat{p}_{\mathrm{I}}$ ($\hat{p}_{\mathrm{II}}$) represents the annihilation operator of the branch I (II), and $\hat{p}_{\mathrm{I}}^{\dagger}$ ($\hat{p}_{\mathrm{II}}^{\dagger}$) represents the creation operator of the branch I (II). The new magnon-magnon polaron Boson operators can be expressed in terms of the magnon Boson operators:

$$\begin{bmatrix} \hat{p}_{\mathrm{I}} \\ \hat{p}_{\mathrm{II}} \\ \hat{p}_{\mathrm{I}}^{\dagger} \\ \hat{p}_{\mathrm{II}}^{\dagger} \end{bmatrix} = \begin{bmatrix} w_{\mathrm{I}} & x_{\mathrm{I}} & y_{\mathrm{I}} & z_{\mathrm{I}} \\ w_{\mathrm{II}} & x_{\mathrm{II}} & y_{\mathrm{II}} & z_{\mathrm{II}} \\ y_{\mathrm{I}}^{*} & z_{\mathrm{I}}^{*} & w_{\mathrm{I}}^{*} & x_{\mathrm{I}}^{*} \\ y_{\mathrm{II}}^{*} & z_{\mathrm{II}}^{*} & w_{\mathrm{II}}^{*} & x_{\mathrm{II}}^{*} \end{bmatrix} \begin{bmatrix} \hat{a}_{+} \\ \hat{a}_{-} \\ \hat{a}_{+}^{\dagger} \\ \hat{a}_{-}^{\dagger} \end{bmatrix} \tag{S-21}$$

where

$$w_{\mathrm{i}}w_{\mathrm{j}}^{*} + x_{\mathrm{i}}x_{\mathrm{j}}^{*} - y_{\mathrm{i}}y_{\mathrm{j}}^{*} - z_{\mathrm{i}}z_{\mathrm{j}}^{*} = \delta_{\mathrm{ij}},\ i(j) \in \{\mathrm{I}, \mathrm{II}\}$$

In order to figure out the coefficients and the frequencies of the two new modes, we solve the Heisenberg equations of motion

$$\omega_{\mathrm{I(II)}} \begin{bmatrix} w_{\mathrm{I(II)}} \\ x_{\mathrm{I(II)}} \\ y_{\mathrm{I(II)}} \\ z_{\mathrm{I(II)}} \end{bmatrix} = \begin{bmatrix} \omega_{+} & \mathrm{i}g_1 & 0 & \mathrm{i}g_2 \\ -\mathrm{i}g_1 & \omega_{-} & \mathrm{i}g_2 & 0 \\ 0 & \mathrm{i}g_2 & -\omega_{+} & \mathrm{i}g_1 \\ \mathrm{i}g_2 & 0 & -\mathrm{i}g_1 & -\omega_{-} \end{bmatrix} \begin{bmatrix} w_{\mathrm{I(II)}} \\ x_{\mathrm{I(II)}} \\ y_{\mathrm{I(II)}} \\ z_{\mathrm{I(II)}} \end{bmatrix} \tag{S-22}$$

By solving this eigenvalue equation set, the general solution of the $(w_{\mathrm{I(II)}}, x_{\mathrm{I(II)}}, y_{\mathrm{I(II)}}, z_{\mathrm{I(II)}})^{\mathrm{T}}$ and $\omega_{\mathrm{I(II)}}$ can be obtained. We display the expression of $\omega_{\mathrm{I(II)}}$ below

$$\omega_{\mathrm{I(II)}}^2 = \frac{1}{2}\left[2g_1^2 - 2g_2^2 + \omega_+^2 + \omega_-^2 \pm \sqrt{\left(\omega_+^2 - \omega_-^2\right)^2 + 4g_1^2\left(\omega_+ + \omega_-\right)^2 - 4g_2^2\left(\omega_+ - \omega_-\right)^2}\right] \quad \text{(S-23)}$$

Equation S-20 corresponds to a diagonalized Hamiltonian. Its eigenstates can be expressed as $\left|n_{\mathrm{I}}, m_{\mathrm{II}}\right\rangle_{\mathrm{c}}$, $n(m) \in \{0,1,2\cdots\}$, where n_{I} (m_{II}) represents the number of magnon-magnon polaron of the branch I (II). When n_{I} and m_{II} are equal to zero, the eigenstate corresponds to the ground state. By applying $\hat{p}_{\mathrm{I(II)}}$ on the ground state, we obtain

$$\hat{p}_{\mathrm{I(II)}}\left|0_{\mathrm{I}}, 0_{\mathrm{II}}\right\rangle_{\mathrm{c}} = \left(w_{\mathrm{I(II)}}\hat{a}_+ + x_{\mathrm{I(II)}}\hat{a}_- + y_{\mathrm{I(II)}}\hat{a}_+^\dagger + z_{\mathrm{I(II)}}\hat{a}_-^\dagger\right)\left|0_{\mathrm{I}}, 0_{\mathrm{II}}\right\rangle_{\mathrm{c}} = 0 \quad \text{(S-24)}$$

It can be seen that when $g_2 \neq 0$, the polaron annihilation operator can be expressed as a linear superposition of the annihilation operators and creation operators of the original + mode and − mode, so the ground state of the coupled Hamiltonian is a squeezed vacuum of the + mode and − mode magnons. Such a squeezed vacuum can result in a reduced quantum fluctuation and a non-zero magnon number.

We first define the following quadrature

$$\hat{X}_{\phi,\psi} = \frac{1}{\sqrt{8}}\left(\hat{a}_+ \mathrm{e}^{-\mathrm{i}\phi} + \hat{a}_+^\dagger \mathrm{e}^{\mathrm{i}\phi} + \hat{a}_- \mathrm{e}^{-\mathrm{i}\psi} + \hat{a}_-^\dagger \mathrm{e}^{\mathrm{i}\psi}\right) \quad \text{(S-25)}$$

where ϕ and ψ correspond to two independent phases. The fluctuation of the quadrature in any quantum state is defined as

$$\left\langle \delta\hat{X}_{\phi,\psi}^2 \right\rangle = \left\langle \left(\hat{X}_{\phi,\psi} - \left\langle \hat{X}_{\phi,\psi} \right\rangle\right)^2 \right\rangle \quad \text{(S-26)}$$

The uncertainty principle requires

$$\left\langle \delta\hat{X}_{\phi,\psi}^2 \right\rangle \cdot \left\langle \delta\hat{X}_{\phi+\pi/2,\psi+\pi/2}^2 \right\rangle \geq \left(\frac{1}{2\mathrm{i}}\left\langle \left[\hat{X}_{\phi,\psi}, \hat{X}_{\phi+\pi/2,\psi+\pi/2}\right] \right\rangle\right)^2 = \frac{1}{16} \quad \text{(S-27)}$$

Therefore, the standard quantum limit for the quadrature is 0.25.

We first consider the quantum fluctuation of the quadrature in the ground state of $\hat{\mathcal{H}}_{\text{Pure}}$

$$ {}_{\text{d}}\left\langle \delta\hat{X}^2_{\phi,\psi} \right\rangle_{\text{d}} = {}_{\text{d}}\left\langle 0_+,0_- \right| \hat{X}^2_{\phi,\psi} \left| 0_+,0_- \right\rangle_{\text{d}} - \left({}_{\text{d}}\left\langle 0_+,0_- \right| \hat{X}_{\phi,\psi} \left| 0_+,0_- \right\rangle_{\text{d}} \right)^2 = \frac{1}{4} \tag{S-28} $$

Therefore, the quantum fluctuation is equal to the standard quantum limit, regardless of the phases ϕ and ψ. And the ground state of $\hat{\mathcal{H}}_{\text{Pure}}$ corresponds to a trivial ground state, where no squeezing effect is observed. The average magnon numbers are zero in this trivial ground state.

Next, we consider the ground state of the full Hamiltonian $\hat{\mathcal{H}}$. The quantum fluctuation of the quadrature in this case is

$$ \begin{aligned} {}_{\text{c}}\left\langle \delta\hat{X}^2_{\phi,\psi} \right\rangle_{\text{c}} &= {}_{\text{c}}\left\langle 0_{\text{I}},0_{\text{II}} \right| \hat{X}^2_{\phi,\psi} \left| 0_{\text{I}},0_{\text{II}} \right\rangle_{\text{c}} - \left({}_{\text{c}}\left\langle 0_{\text{I}},0_{\text{II}} \right| \hat{X}_{\phi,\psi} \left| 0_{\text{I}},0_{\text{II}} \right\rangle_{\text{c}} \right)^2 \\ &= \frac{1}{8}\Big[\left(w_{\text{I}}^* \,\text{e}^{-\text{i}\phi} - y_{\text{I}}^* \,\text{e}^{\text{i}\phi} + x_{\text{I}}^* \,\text{e}^{-\text{i}\psi} - z_{\text{I}}^* \,\text{e}^{\text{i}\psi} \right)\left(-y_{\text{I}} \,\text{e}^{-\text{i}\phi} + w_{\text{I}} \,\text{e}^{\text{i}\phi} - z_{\text{I}} \,\text{e}^{-\text{i}\psi} + x_{\text{I}} \,\text{e}^{\text{i}\psi} \right) + \\ &\quad \left(w_{\text{II}}^* \,\text{e}^{-\text{i}\phi} - y_{\text{II}}^* \,\text{e}^{\text{i}\phi} + x_{\text{II}}^* \,\text{e}^{-\text{i}\psi} - z_{\text{II}}^* \,\text{e}^{\text{i}\psi} \right)\left(-y_{\text{II}} \,\text{e}^{-\text{i}\phi} + w_{\text{II}} \,\text{e}^{\text{i}\phi} - z_{\text{II}} \,\text{e}^{-\text{i}\psi} + x_{\text{II}} \,\text{e}^{\text{i}\psi} \right) \Big] \end{aligned} \tag{S-29} $$

The average magnon numbers in this case are

$$ \begin{aligned} {}_{\text{c}}\left\langle \hat{a}_+^\dagger \hat{a}_+ \right\rangle_{\text{c}} &= y_{\text{I}} y_{\text{I}}^* + y_{\text{II}} y_{\text{II}}^* \\ {}_{\text{c}}\left\langle \hat{a}_-^\dagger \hat{a}_- \right\rangle_{\text{c}} &= z_{\text{I}} z_{\text{I}}^* + z_{\text{II}} z_{\text{II}}^* \end{aligned} \tag{S-30} $$

We will neglect the subscript "c" in the following part.

Now we discuss some specific examples. Figure S8 and Figure S9 display the results of the quantum fluctuations and the average magnon numbers of three typical SAFs, respectively. We first consider the PMA SAF example, where H_{k1} = 0 kOe, H_{k2} = 5 kOe. When $H = H_0 = 0$, $g_2 = 0$. In this case, $\hat{p}_{\text{I(II)}}$ does not contain $\hat{a}_+^\dagger$ and $\hat{a}_-^\dagger$. Therefore, the ground state in this case is the same as the ground state of $\hat{\mathcal{H}}_{\text{Pure}}$. The quantum fluctuation is equal to 0.25 independent of ϕ and ψ, as shown in Figs. S8b and S8c. Thus, the minimum quantum fluctuation is also equal to 0.25, as shown in the violet circle in Fig. S8a. And the average magnon numbers $\left\langle \hat{a}_+^\dagger \hat{a}_+ \right\rangle$ and $\left\langle \hat{a}_-^\dagger \hat{a}_- \right\rangle$ are equal to 0, as shown in Fig. S9a. When $H \neq 0$, g_2 is no longer equal to 0. Take H = 4 kOe for example, which represents the general case in PMA SAF. $\hat{p}_{\text{I(II)}}$ in this case contains all the four annihilation operators and creation operators, as shown in Eq. S-24. Therefore, squeezing effect

occurs. In Figs. S8d and S8e, we display the quantum fluctuation $\left\langle \delta\hat{X}^2_{\phi,\psi} \right\rangle$ as a function of phases ϕ and ψ, and as a function of ϕ when $\phi = \psi$, respectively. The results show that at some (ϕ, ψ), the quantum fluctuation decreases, while at the other (ϕ, ψ), the quantum fluctuation increases, since the uncertainty principle needs to be satisfied. The minimum quantum fluctuation is shown in the orange circle in Fig. S8a. And the average magnon numbers are not equal to 0, as shown in the orange circle in Fig. S9a. But due to the small g_2 in this case, the squeezing effect is weak and the average magnon numbers are small.

We then consider the IP SAF example, where H_{k1} = 0 kOe, H_{k2} = −5 kOe. We note that when H approaches 0, $\hat{p}_{\mathrm{I(II)}}$ can be expressed as

$$\begin{aligned} \hat{p}_{\mathrm{I}} &= \hat{a}_+ \\ \hat{p}_{\mathrm{II}} &= \cosh r\hat{a}_- + \sinh r\hat{a}_-^\dagger \end{aligned} \tag{S-31}$$

where r is the relevant squeeze parameter. In this case, the ground state is a single-mode squeezed vacuum of the − mode magnon. We define the following − mode magnon squeeze operator

$$\hat{S}_- = \exp\left(\frac{r}{2}\hat{a}_-^2 - \frac{r}{2}\hat{a}_-^{\dagger 2}\right) \tag{S-32}$$

We have

$$\left|0_{\mathrm{I}}, 0_{\mathrm{II}}\right\rangle_{\mathrm{c}} = \hat{S}_- \left|0_+, 0_-\right\rangle_{\mathrm{d}} \tag{S-33}$$

In this case, the quantum fluctuation is independent of ϕ:

$$\left\langle \delta\hat{X}^2_{\phi,\psi} \right\rangle = \frac{1}{8}\left(1 + \mathrm{e}^{-2r}\cos^2\psi + \mathrm{e}^{2r}\sin^2\psi\right) \tag{S-34}$$

And the average magnon numbers are

$$\begin{aligned} \left\langle \hat{a}_+^\dagger \hat{a}_+ \right\rangle &= 0 \\ \left\langle \hat{a}_-^\dagger \hat{a}_- \right\rangle &= \sinh^2 r \end{aligned} \tag{S-35}$$

By substituting the specific value of r into Eq. S-34 and Eq. S-35, we can get the results as shown in Figs. S8g, S8h and S9b, and as shown in the violet circle in Fig. S8f. When $H = H_0$, we note that this case possesses maximum squeezing effect for IP SAF, as shown in the orange circle in Fig. S8f. The results of the quantum fluctuation and the average magnon numbers are displayed in Figs. S8i, S8j and S9b. We note that in this case, the matrix elements in Eq. S-21 satisfy the following relations: $x_{\mathrm{I}} = -\mathrm{i}w_{\mathrm{I}}$, $z_{\mathrm{I}} = \mathrm{i}y_{\mathrm{I}}$, $x_{\mathrm{II}} = \mathrm{i}w_{\mathrm{II}}, z_{\mathrm{II}} = -\mathrm{i}y_{\mathrm{II}}$, which causes $\left\langle \delta\hat{X}^2_{\phi,\phi} \right\rangle$ to be symmetric about the 45° axis and $\left\langle \hat{a}_+^\dagger \hat{a}_+ \right\rangle$ being equal to $\left\langle \hat{a}_-^\dagger \hat{a}_- \right\rangle$.

Finally, we consider the T-Type example, where $H_{\mathrm{k1}} = -3$ kOe, $H_{\mathrm{k2}} = 5$ kOe. We note that when H approaches 0, the SAF system approaches the superradiant phase transition, which leads to nearly zero quantum fluctuation and macroscopic excitations in the ground state, as shown in Figs. S8k and S9c. To prevent divergency, we study the quantum fluctuation when H is tiny, as shown in Figs. S8l and S8m. Figure S8l shows completely different features from the above-mentioned plots and a shape similar to "8" is obtained in Fig. S8m, where the minimum is obtained near 45°. As mentioned in the main text, we can realize pure counter-rotating coupling in our T-Type configuration. Here, we study the ground-state properties in this case. We note that when $g_{\mathrm{I}} = 0$, $\hat{p}_{\mathrm{I}}$ and $\hat{p}_{\mathrm{II}}$ can be expressed as

$$\begin{aligned} \hat{p}_{\mathrm{I}} &= x_{\mathrm{I}}\hat{a}_- + y_{\mathrm{I}}\hat{a}_+^\dagger = \cosh r_0 \hat{a}_- + \mathrm{i}\sinh r_0 \hat{a}_+^\dagger \\ \hat{p}_{\mathrm{II}} &= w_{\mathrm{II}}\hat{a}_+ + z_{\mathrm{II}}\hat{a}_-^\dagger = \cosh r_0 \hat{a}_+ + \mathrm{i}\sinh r_0 \hat{a}_-^\dagger \end{aligned} \qquad \text{(S-36)}$$

where r_0 is the relevant squeeze parameter. In this case, the ground state is a standard two-mode squeezed vacuum. We define the following two-mode squeeze operator

$$\hat{S}_2 = \exp\left(-\mathrm{i}\, r_0 \hat{a}_+ \hat{a}_- - \mathrm{i}\, r_0 \hat{a}_+^\dagger \hat{a}_-^\dagger\right) \qquad \text{(S-37)}$$

We have

$$\left|0_{\mathrm{I}}, 0_{\mathrm{II}}\right\rangle_{\mathrm{c}} = \hat{S}_2 \left|0_+, 0_-\right\rangle_{\mathrm{d}} \qquad \text{(S-38)}$$

In this case, when $\phi = \psi$, the quantum fluctuation as a function of ϕ is

$$\left\langle \delta\hat{X}^2_{\phi,\phi} \right\rangle = \frac{\mathrm{e}^{2r_0}}{4}\sin^2\left(\frac{\pi}{4} - \phi\right) + \frac{\mathrm{e}^{-2r_0}}{4}\cos^2\left(\frac{\pi}{4} - \phi\right) \qquad \text{(S-39)}$$

And the average magnon numbers are

$$\left\langle \hat{a}_+^\dagger \hat{a}_+ \right\rangle = \left\langle \hat{a}_-^\dagger \hat{a}_- \right\rangle = \sinh^2 r_0 \quad \text{(S-40)}$$

By substituting the specific value of r_0 into Eq. S-39 and Eq. S-40, we can get the results as shown in Figs. S8n and S8o, and as shown in the orange circles in Figs. S8k and S9c.

Section S8. Calculated squeezed quantum fluctuations and average magnon numbers when anisotropic asymmetry changes

As shown in Fig. S10, we draw the color plots of calculated average + mode magnon number (Fig. S10a) and minimum quantum fluctuation (Fig. S10b) at H_0. $\log\left(\left\langle \hat{a}_+^\dagger \hat{a}_+ \right\rangle + 1\right)$ is displayed for clarity in Fig. S10a. Similarly, grey dashed curves in each plot indicate the boundary between two different magnetization configurations. It can be seen that quite different results are obtained in different configuration regions. In the PMA SAF region, average + mode magnon number and minimum quantum fluctuation are strictly equal to 0 and 0.25, respectively, which means that the ground state of PMA SAF is a trivial ground state without squeezing effect when $H = H_0$. This is because the coupling in PMA SAF is totally co-rotating at H_0, which preserves the particle population. Therefore, the vacuum is not modified by the coupling term and remains trivial even when the coupling strength is large. In the IP SAF region, $\left\langle \hat{a}_+^\dagger \hat{a}_+ \right\rangle$ and $\min\left(\left\langle \delta\hat{X}_{\phi,\psi}^2 \right\rangle\right)$ are equal to the case of trivial ground state only when $H_{k1} = H_{k2}$. As the system deviates from the symmetric position, $\left\langle \hat{a}_+^\dagger \hat{a}_+ \right\rangle$ increases from 0 and $\min\left(\left\langle \delta\hat{X}_{\phi,\psi}^2 \right\rangle\right)$ decreases from the standard quantum limit, which indicates that the squeezed vacuum is formed and magnonic excitations are contained in the ground state since g_2 is no longer equal to 0. In the T-Type regions, as mentioned in Fig. 1 in the main text, the ω_{II} at H_0 is equal to 0. We note that this phenomenon is a feature of the superradiant phase transition. Therefore, the superradiant phase transition occurs in our T-Type configuration at H_0, which leads to macroscopic excitations in the ground state. However, in this case, the average magnon number diverges based on the quadratic Hopfield model. Thus, to avoid the divergence, we consider a slight 10^{-5} Oe deviation from H_0 when we calculate, which can also capture the characteristics of the T-Type regions. The results show that a large average magnon number exists in the ground state of T-Type when approaching the superradiant phase transition, which is usually several orders of magnitude larger than the average magnon number in IP SAF. More interestingly, a nearly zero quantum fluctuation is obtained in the T-Type

regions, which is also associated with the superradiant phase transition. The above discussion is based on the interior of each region. We note that some interesting phenomena are observed in the vicinity of the boundaries. As labeled by the green arrows in each plot, we choose two typical cases, where H_{k2} = 5 kOe (T1) and −5 kOe (T2). The cross sections are shown on the right. We first consider the T1 case, where PMA SAF transforms to T-Type with the decrease of H_{k1}. It can be seen that $\langle \hat{a}_+^\dagger \hat{a}_+ \rangle$ shows a sudden jump when this transformation occurs and the minimum quantum fluctuation shows a sudden drop from the standard quantum limit to nearly 0. We point out that the particular coupling property of our SAF system plays an extremely important role in inducing these PMA SAF-T-Type boundary effects. As mentioned in Fig. 2b in the main text, g_2 is strictly equal to 0 in the PMA SAF region, which means the trivial ground state property is preserved in PMA SAF even when approaching the superradiant phase transition. However, as long as the configuration transforms to T-Type, g_2 emerges, which immediately leads to the rapid modification of the ground state. Therefore, by tuning the magnetic anisotropy near the PMA SAF-T-Type boundary, the nontrivial property of the ground state can be switched on or off. For the T2 case, IP SAF transforms to T-Type with the increase of H_{k1}. We note that the boundary shows extremely different properties with the T1 case. When approaching the boundary from the IP SAF side, $\langle \hat{a}_+^\dagger \hat{a}_+ \rangle$ diverges and the minimum quantum fluctuation approaches 0. While $\langle \hat{a}_+^\dagger \hat{a}_+ \rangle$ shows a decrease feature and the minimum quantum fluctuation increases when approaching the boundary from the T-Type side. The reason is that when approaching the boundary, the ω_{II} of IP SAF approaches 0, which leads to the occurrence of the superradiant phase transition. While g_1/ω_0 and g_2/ω_0 of T-Type decrease to nearly 0 as shown in Fig. 2a in the main text, which results in only a slight modification of the ground state even when the deviation from H_0 is small.

Section S9. Examples of calculated VRSSs, VBSSs and ground-state energy in SAF system

Figures S11a to S11c and Figures S12a to S12c display the results of the vacuum Rabi splitting-induced shifts (VRSSs), the vacuum Bloch-Siegert shifts (VBSSs) and the ground-state energy of three typical SAFs, respectively. Three typical SAFs are PMA SAF (H_{k1} = 0 kOe, H_{k1} = 5 kOe), T-Type (H_{k1} = −10 kOe, H_{k1} = 5 kOe) and IP SAF (H_{k1} = 0 kOe, H_{k1} = −5 kOe), respectively. We first illustrate the VRSSs and VBSSs. As shown in Figs. S11a to S11c, in each plot, we use the gray dashed curves to represent the pure − and + modes, the black solid curves to represent the branch I and branch II, and the red curves to represent the co-rotating coupled

branches where we set g_2 to be 0 on purpose. The VRSSs are indicated by the differences between red solid curves and gray dashed curves, while the VBSSs are indicated by the differences between red solid curves and black solid curves, which are labelled by the violet arrows. Take the branch I as an example, we display the corresponding extracted VRSS and VBSS as functions of H in the inset. We then illustrate the ground-state energy. Considering the full Hamiltonian $\hat{\mathcal{H}}$ and the pure Hamiltonian $\hat{\mathcal{H}}_{\text{Pure}}$ displayed in Eq. 1 and Eq. S-18, we note that the ground-state energy in these two cases can be written as $(1/2)\hbar(\omega_{\text{I}}+\omega_{\text{II}})$ and $(1/2)\hbar(\omega_{+}+\omega_{-})$, respectively. We use $\Delta E_{\text{G}}/E_{\text{G}}$ to indicate the change of the ground-state energy E_{G} when the coupling terms are added to $\hat{\mathcal{H}}_{\text{Pure}}$, which is defined as

$$\frac{\Delta E_{\text{G}}}{E_{\text{G}}}=\frac{\frac{1}{2}\hbar(\omega_{\text{I}}+\omega_{\text{II}})-\frac{1}{2}\hbar(\omega_{+}+\omega_{-})}{\frac{1}{2}\hbar(\omega_{+}+\omega_{-})}=\frac{\omega_{\text{I}}+\omega_{\text{II}}}{\omega_{+}+\omega_{-}}-1 \tag{S-41}$$

Therefore, the change in the ground-state energy is reflected in the change in spectra. Thus, the ground-state energy is relevant to the VRSSs and VBSSs.

For PMA SAF example, we note that when $H = H_0 = 0$, only VRSS exists as shown in the inset of Fig. S11a due to the zero g_2 in this case. With the increase of H, the VBSS is no longer equal to 0 and the VRSS and VBSS show nonmonotonic feature. Note that the VRSS decreases to 0 at the H with pure counter-rotating coupling, which indicates that we can realize pure VBSS in our SAF system. And the ground-state energy is unaltered when $H = H_0 = 0$. When $H \neq 0$, the ground-state energy decreases, and the value of $\Delta E_{\text{G}}/E_{\text{G}}$ first decreases and then increases with the increase of H.

For T-Type example, when $H = H_0 = 0$, the VBSS is larger than the VRSS due to the large g_2 which is more than twice as much as g_1 in this case. Note that we can also realize pure VBSS in this case at the H with pure counter-rotating coupling, but the VBSS is much larger than the PMA SAF case. Due to the large VBSS, the ground-state energy is greatly reduced when $H = H_0 = 0$, and $\Delta E_{\text{G}}/E_{\text{G}}$ is around −0.4. With the increase of H, $\Delta E_{\text{G}}/E_{\text{G}}$ first increases, then decreases and finally increases.

For IP SAF example, when $H = H_0$, the VRSS reaches the peak, which has the maximum value. And the VBSS is not equal to 0. But since g_2 is equal to g_1 in the IP SAF case, the VBSS is much

smaller than the VRSS. And $\Delta E_G/E_G$ reaches the minimum value when $H = H_0$. Due to the relatively small g_2, this minimum is much larger than the minimum in our T-Type example.

We give the VBSSs, the VRSSs and $\Delta E_G/E_G$ as functions of the magnetic anisotropy H_{k1} at H_0 in Figs. S11d, S11e and Figs. S12d, S12e, respectively. The results show agreement with Figs. S11a to S11c and Figs. S12a to S12c. In PMA SAF region, the VBSS(H_0) and $\Delta E_G/E_G(H_0)$ are all equal to 0. And the VRSS(H_0) increases with the increase of anisotropic asymmetry. In IP SAF region, the VBSS(H_0) is much smaller than VRSS(H_0). And the absolute value of $\Delta E_G/E_G(H_0)$ is small in most cases. But $\Delta E_G/E_G(H_0)$ shows a sharp decrease when H_{k1} approaches the boundary, where the superradiant phase transition occurs. In T-Type region, the VBSS(H_0) can be smaller or larger than VRSS(H_0) depending on the choice of H_{k1}. We use gray color to label the regions with VBSS(H_0) > VRSS(H_0), where g_2 is much larger than g_1. And the absolute value of $\Delta E_G/E_G(H_0)$ can be large when VBSS(H_0) > VRSS(H_0) is satisfied.

Section S10. The effect of different initial configurations of SAFs

We take a typical PMA SAF example to illustrate the effect of different initial configurations on the resonance properties in this section. Figure S13a schematically shows two initial configurations for PMA SAF: "head-to-head" and "tail-to-tail". In this section, θ_1 and θ_2 represent the equilibrium polar angles of $\mathbf{m_1}$ and $\mathbf{m_2}$. Head-to-head configuration means $\theta_1 = 0°$, $\theta_2 = 180°$ when $H = 0$. And tail-to-tail configuration means $\theta_1 = 180°$, $\theta_2 = 0°$ when $H = 0$. The resonance spectra of these two configurations are obtained by micromagnetic simulation (color) and theoretical calculation (curves), as shown in Figs. S13b and S13c. From these two plots, it is found that there is no difference in spectra between the two configurations. Figures S13d and S13e show the simulated corresponding equilibrium positions of $\mathbf{m_1}$ and $\mathbf{m_2}$. We use $\theta_{1(H)}$ and $\theta_{2(H)}$ to represent the equilibrium angles obtained in head-to-head configuration, and $\theta_{1(T)}$ and $\theta_{2(T)}$ to represent the equilibrium angles obtained in tail-to-tail configuration. They satisfy the following relations: $\theta_{1(H)} + \theta_{1(T)} = 180°$ and $\theta_{2(H)} + \theta_{2(T)} = 180°$. Figures S13f and S13g further show the magnetization oscillations in the time domain for different initial configurations. For convenience, we define the right-hand (RH) polarization and left-hand (LH) polarization for a single magnetic moment $\mathbf{m_1}$ ($\mathbf{m_2}$). The RH (LH) polarization means the magnetic moment precesses counter-clockwise (clockwise) around its equilibrium position, as schematically shown in Fig. S13f. For the case of $H = 1$ kOe, $\mathbf{m_1}$ and $\mathbf{m_2}$ process the LH (RH) polarization and RH

(LH) polarization when the branch I (II) is excited, respectively. We find that the polarization for a single magnetic moment is independent of the initial configurations. Besides, the amplitude of oscillation of $\mathbf{m}_1$ ($\mathbf{m}_2$) is also independent of the initial configurations. The study for the case of higher H shows that, the polarization of $\mathbf{m}_1$ ($\mathbf{m}_2$) becomes RH when the branch I (II) is excited, which is also independent of the initial configurations. And the amplitude of oscillation of $\mathbf{m}_1$ ($\mathbf{m}_2$) is still independent of the initial configurations. We also study the effect of the initial configuration on T-Type, and we find that all the properties mentioned above are satisfied in T-Type, except that only the RH polarization is observed in $\mathbf{m}_1$ ($\mathbf{m}_2$). Therefore, their spectra are independent of the initial configurations when H is applied in the SAF plane for both PMA SAF and T-Type structures. And when one of the branches exists, $\mathbf{m}_1$ ($\mathbf{m}_2$) of the two initial configurations are symmetric with respect to the x axis (the direction of H) at any time. We point out that this is because H is applied in the plane, and the total effective fields of $\mathbf{m}_1$ ($\mathbf{m}_2$) in both initial configurations are equivalent.

Section S11. Effect of the biquadratic exchange interaction and asymmetry of M_S and d between FM1 and FM2 on coupling properties

We show three examples to illustrate the effect of the biquadratic exchange interaction and asymmetry of M_S and d between FM1 and FM2 on coupling properties. As shown in Fig. S14, we calculate the g_1/ω_0 and g_2/ω_0 as functions of ξ and κ' based on the complete case. ξ is defined as $\xi = H_{\text{ex}2}^{(1)} \big/ H_{\text{ex}1}^{(1)}$, which is the ratio of the biquadratic exchange field to the bilinear exchange field. κ' is defined as $\kappa' = \left(M_{S1}d_1 - M_{S2}d_2\right) \big/ \left(M_{S1}d_1 + M_{S2}d_2\right)$, which represents the degree of asymmetry of the magnetic moments.

We first consider the PMA SAF example, as shown in Figs. S14a and S14b. In this example, the H_{k1}, H_{k2} and $\overline{H}_{\text{ex}1} = (H_{\text{ex}1}^{(1)} + H_{\text{ex}1}^{(2)})/2$ are set to the same as the samples S1 in order to compare with the experiment. It can be seen that when ξ is not large, g_1/ω_0 increases with the decrease of κ' and with the increase of ξ, while g_2/ω_0 stays at 0. However, when ξ goes up to a certain point, the antiparallel configuration of the two magnetic moments cannot be maintained at $H = 0$ and the system changes to T-Type. In this case, g_2/ω_0 is no longer equal to 0 and increases with the increase of ξ, while g_1/ω_0 decreases with the increase of ξ. We use circles to label the corresponding parameters of S1, which indicates that we can further increase g_1/ω_0 of S1 by

decreasing κ' or (and) increasing ξ. Compared with the $\kappa' = 0$ and $\xi = 0$ case, we note that the non-zero ξ and κ' result in 17% g_1/ω_0 change in the sample S1.

We then consider the T-Type example, as shown in Figs. S14c and S14d. In this example, the H_{k1}, H_{k2} and $\bar{H}_{ex1} = (H_{ex1}^{(1)} + H_{ex1}^{(2)})/2$ are set to the same as the samples S2. It can be seen that both g_1/ω_0 and g_2/ω_0 increase with the increase of ξ. And with the increase of κ', the influence of the change of ξ on g_1/ω_0 gradually decreases, while the influence of the change of ξ on g_2/ω_0 gradually increases. The corresponding parameters of S2 are marked by the circles. Thus, to further increase g_1/ω_0 (g_2/ω_0), we can decrease (increase) κ' and increase ξ. Compared with the $\kappa' = 0$ and $\xi = 0$ case, we note that the non-zero ξ and κ' result in 7.7% g_1/ω_0 change and 2.3% g_2/ω_0 change in the sample S2.

Finally, we consider the IP SAF example, as shown in Figs. S14e and S14f. In this example, the H_{k1}, H_{k2} and $\bar{H}_{ex1} = (H_{ex1}^{(1)} + H_{ex1}^{(2)})/2$ are set to the same as the samples S3. It can be seen that when the value of ξ is small, g_1/ω_0 increases with the decrease of κ', and g_2/ω_0 increases with the increase of κ'. With the increase of ξ, the influence of the change of κ' on g_1/ω_0 and g_2/ω_0 gradually decreases. The corresponding parameters of S3 are marked by the circles. Thus, to further increase g_1/ω_0 (g_2/ω_0), we can decrease (increase) κ' and decrease ξ. Compared with the $\kappa' = 0$ and $\xi = 0$ case, we note that the non-zero ξ and κ' result in −15% g_1/ω_0 change and 5.9% g_2/ω_0 change in the sample S3.

Section S12. Detailed fitting process and comparison of resonance spectrum fitting results with VSM fitting results

We take the fitting process and results from S1 to S3 as an example. The fittings are based on the complete case, where six parameters: H_{ki}, $H_{ex1}^{(i)}$, $H_{ex2}^{(i)}$ (i = 1, 2), need to be determined in order to get certain branch I and branch II, as shown in Eq. 6, Section S3, and Eq. S-23. Here, i = 1 and 2 refer to FM1 and FM2, respectively. We point out that it is improper to determine all parameters in a single fitting, because some combinations of completely different values of parameters may yield similar results, which may reduce the reliability of the fitting. To obtain convincing fitting results, some parameters need to be fixed beforehand. We propose that H_{k1}, H_{k2} and $H_{ex1}^{(2)}/H_{ex1}^{(1)} = M_{S1}d_1/M_{S2}d_2$ can be obtained by fitting the hysteresis loops of single FMs with the same structures of FMi (i = 1, 2) in SAFs. Hence, five samples with single FM are prepared for fitting: Ta /Pt /[Co (0.4)/Ni (0.8)]$_3$/Co (0.4)/Ir /Ta, Ta /Pt /Ir (0.64)/[Co (0.64)/Ni

(1.28)]$_2$/Co (0.64)/Pt /Ta, Ta /Pt /Ir (0.6)/[Co (0.4)/Ni (0.8)]$_3$/Co (0.4)/Ir /Ta, Ta /Pt /Ir (0.6)/[Co (0.68)/Ni (1.36)]$_2$/Co (0.68)/Ir /Ta and Ta /Ir /$Ni_{80}Fe_{20}$ (3)/Pt /Ta, where the thicknesses in brackets are given in nanometer. By fitting the hysteresis loops, H_k for these five samples are determined to be 4900, 690, 3720, 750 and −9570 Oe, respectively. Similarly, we can also extract M_S and d of each sample. Therefore, for S1, S2 and S3, $H_{ex1}^{(2)}/H_{ex1}^{(1)}$ are determined to be 0.79, 1.30 and 1.40, respectively. We assume that the magnetic anisotropy of one FM in a SAF is approximately equal to that obtained in a single FM with the same structure, except for the case of PMA SAF. In the PMA SAF sample S1, the magnetic anisotropy of upper Co/Ni stack is strongly influenced by the underlying structure. Hence, we obtain H_{k2} in S1 by fitting the hysteresis loop of the entire sample, and then we fix it when fit the resonance spectra. Besides, $H_{ex1}^{(i)}$ and $H_{ex2}^{(i)}$ are related by $H_{ex2}^{(2)} = H_{ex2}^{(1)}\left(H_{ex1}^{(2)}/H_{ex1}^{(1)}\right)$.Therefore, only two parameters need to be fitted in the end: $H_{ex1}^{(1)}$ and $H_{ex2}^{(1)}$, so that convinced fitting results can be obtained. Fitting curves of the resonance spectra are shown in Figs. 4a to 4c. And fitting curves of the hysteresis loops are shown in Fig. S15. Table S1 summarizes the parameters obtained by fitting and the corresponding coupling strengths, where the parameters obtained by fitting the resonance spectra are consistent with those obtained by fitting the hysteresis loops.

Section S13. BLS measurement setup

The setup of Brillouin light scattering (BLS) measurement is shown in Fig. S16, where we collect backscattered light and the wavelength λ of the incident light is 532 nm. In our experiment, the angle θ_L of incidence is 0, and the wave vector k = 0 obtained from the relationship $k = 4\pi \times \sin\theta_L/\lambda$. The applied dc magnetic field **H** in the plane is perpendicular to the incidence plane of the light, which corresponds to the Damon-Eshbach spin wave configuration.

Section S14. Details of the spectra shown in Fig. 4

In this section, we add more details about the color plots in Fig. 4. As shown in Figs. S17a to S17c, we select some typical V_{mix}-f spectra from the color plots for samples S1 to S3. These selected spectra are measured at different H, which can well capture the features of the branch I and branch II. The solid curve(s) in each spectrum correspond(s) to the Lorentzian fitting result of experimental data. For IP SAF sample S3, at any H, the branch I and branch II can be distinguished separately, and they are far apart in spectra. Therefore, we fit the branch I and branch II separately for S3. While for the other two samples, at some H, the branch I and branch

II are very close to each other in the spectra. Therefore, the multi-Lorentzian function Eq. 10 is adopted to fit the experimental data for S1 and S2. The spectrum at H_0 for each sample is also displayed. For S3, S1 and S2, H_0 = 1.9, 0, 0 kOe, respectively. Since the ST-FMR measurement requires oscillation of resistance due to the anisotropy magnetoresistance (AMR) or spin Hall magnetoresistance (SMR) effect, we actually add a very small H (~20 Oe) to the samples for the cases of $H \approx 0$ kOe. Figures S17d to S17f show the spectra measured from the BLS method. In these spectra, both negative frequency peaks and positive frequency peaks can be obtained, where the negative frequency and positive frequency peaks correspond to the Stokes and anti-Stokes modes, respectively. The orange curves correspond to the fitting results, and the violet dashed lines indicate the extracted resonance frequencies. In Figs. S17g to S17i, we display the experimentally extracted resonance frequencies from the ST-FMR and BLS measurements as well as the resonance spectra fitted by our theory. Since the Stokes modes and anti-Stokes modes have almost the same resonance frequency, we only show the result of Stokes modes. From these plots, we show that the resonance frequencies obtained by the two techniques are consistent. And the fittings can well reproduce the experimental data, which illustrates the reliability of our theory.

Section S15. Demonstration of the effect of sample volume on the coupling strength

A big difference between magnon-magnon hybrid systems and magnon-photon hybrid systems is that magnon-magnon hybrid systems can be scaled down while maintaining the coupling strength. To prove that, in this section, we take S2 as an example to demonstrate the effect of sample volume on coupling strength. We prepare several samples with the same film structure as S2. The ST-FMR rectangular strips of these samples differ in width: 20, 15, 10, 2 and 0.8 μm, with a constant ratio of length to width. In the ST-FMR experiment in this section, we do not compensate for the loss of microwave power at different frequencies. Thus, in this section, we perform field-sweeping ST-FMR measurement to obtain the resonance fields at different f. And then the coupling strengths of the samples can be extracted by fitting experimental data. The resonance spectra and the fitting curves are displayed in Figs. S19a to S19e, where we use larger microwave power outputs than that in the main text. We note that all these plots show very similar characteristics. g_1/ω_0 and g_2/ω_0 extracted from these samples are shown in Fig. S19f, where the results of sample S2 in the main text are also added for comparison. We note that the

relationship $g_{1(2)}/\omega_0$-width can be fitted with a horizontal line, indicating that the coupling strength is independent of the sample volume in the scale of our experiments.

Section S16. Effect of temperature on the coupling strength

In this section, we demonstrate the effect of temperature on the coupling strength. We take sample S1 as an example and perform field-sweeping ST-FMR measurement in the physical property measurement system (PPMS), which can provide cryogenic environment. The measured resonance spectra are displayed in Figs. S20a to S20c, where the temperature T is set to 10 K, 100 K, and 200 K, respectively. And the curves in each plot correspond to the fitting results. The deviation of the fitted branch II from the experiment at large H is caused by a small tilt of **H** out of the sample plane in the PPMS. We note that with the decrease of temperature, $\omega_{\mathrm{I}}(H_0)$ shows a large increase, while the change of $\omega_{\mathrm{II}}(H_0)$ is not obvious. Therefore, a largely enhanced splitting can be obtained at H_0 by decreasing T. The main reason for this phenomenon is that the magnetic anisotropy of different magnetic layers has different sensitivity to T. By cooling, the PMA of FM1 in S1 is greatly enhanced, which is verified by hysteresis loop measurement of single FM1 in the PPMS. While H_{k2} in S1 does not change significantly with T due to the relatively weak interface magnetic anisotropy. Figure S20d shows the extracted $g_1/2\pi(H_0)$ and g_1/ω_0. Since g_2/ω_0 = 0 in PMA SAF, only $g_1/2\pi(H_0)$ and g_1/ω_0 are displayed. From this plot, it can be seen that $g_1/2\pi(H_0)$ increases with the decrease of T, which shows a large modification of about 40% compared with the $g_1/2\pi(H_0)$ measured at room temperature. While g_1/ω_0 changes slightly with the decrease of T, which is consistent with the discussion of $\omega_{\mathrm{I}}(H_0)$ and $\omega_{\mathrm{II}}(H_0)$ mentioned above.

Reference

1. Holstein T, Primakoff H, Field Dependence of the Intrinsic Domain Magnetization of a Ferromagnet. *Phys Rev* **58**, 1098-1113 (1940).

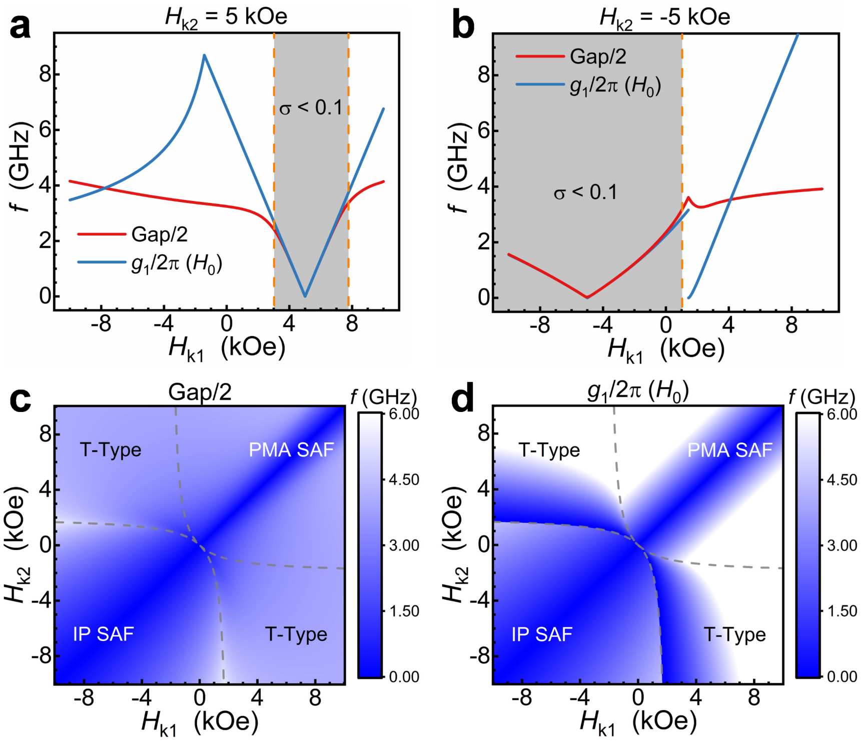

Fig. S1.

Comparison of the coupling strength g_1 and the gap between the two branches. a and **b** $g_1/2\pi$ (H_0) and half of the gap gap/2 as functions of the total magnetic anisotropy field for the lower ferromagnetic layer (FM1) H_{k1}, when the total magnetic anisotropy field for the upper ferromagnetic layer (FM2) H_{k2} = 5 kOe (**a**) and −5 kOe (**b**). Grey regions indicate deviation factor σ less than 0.1. **c** and **d** Color plots of gap/2 (**c**) and $g_1/2\pi$ (H_0) (**d**) as functions of H_{k1} and H_{k2}. The grey dashed curves are defined the same as that in Figs. 2c and 2d.

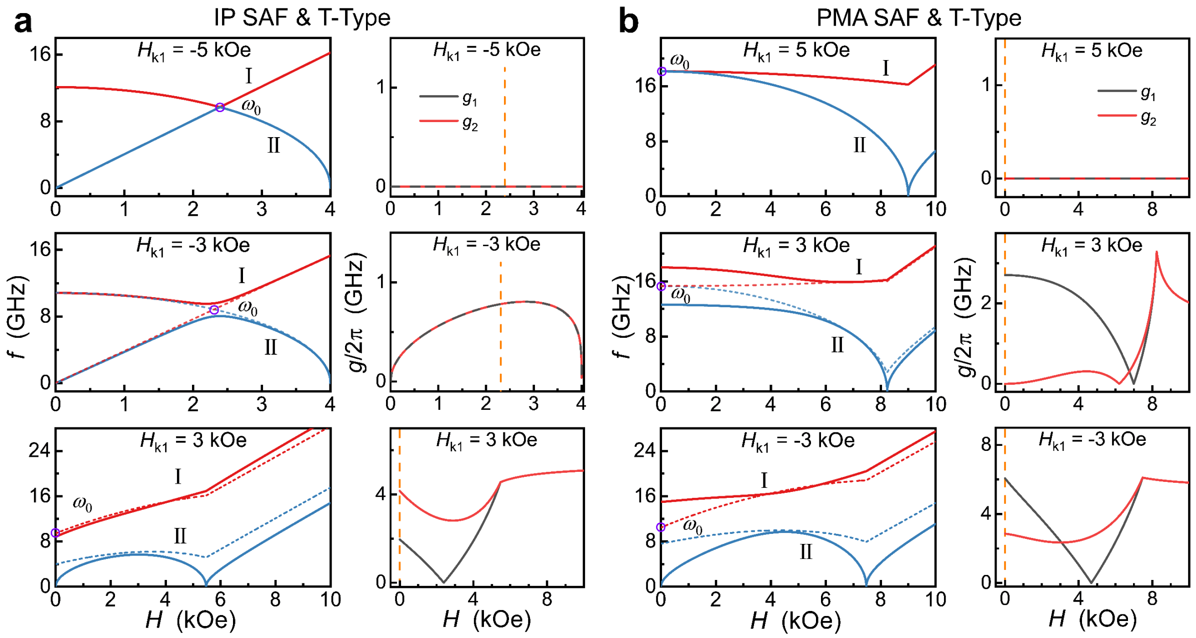

Fig. S2.

Several examples of the resonance frequency and coupling strengths as functions of magnetic field. a and **b** Typical examples of calculated frequency-field dispersions and coupling strengths when H_{k2} = −5 kOe (**a**) and 5 kOe (**b**). The Ruderman-Kittel-Kasuya-Yosida (RKKY) interlayer coupling field H_{ex} is set to −2 kOe in both cases. Gyromagnetic ratio $\gamma/2\pi$ is set to 2.7 GHz/kOe. In (**a**), H_{k1} = −5, −3, 3 kOe from top to bottom case, respectively, which corresponds to the transition from in-plane magnetic anisotropy type synthetic antiferromagnet (IP SAF) in the top two cases to T-shaped magnetization type (T-Type) in the bottom case. While in (**b**), H_{k1} = 5, 3, −3 kOe from top to bottom case, respectively, which corresponds to the transition from perpendicular magnetic anisotropy type (PMA) SAF in the top two cases to T-Type in the bottom case. In the frequency-field dispersions in each case, the red and blue solid curves represent the high-frequency branch (I) and the low-frequency branch (II), respectively. And the red and blue dashed curves represent the decoupled − and + modes, respectively. Violet circle indicates the center frequency ω_0. In the $g/2\pi$-field relationship in each case, the orange dashed line indicates the magnetic field H_0 corresponding to the center frequency ω_0.

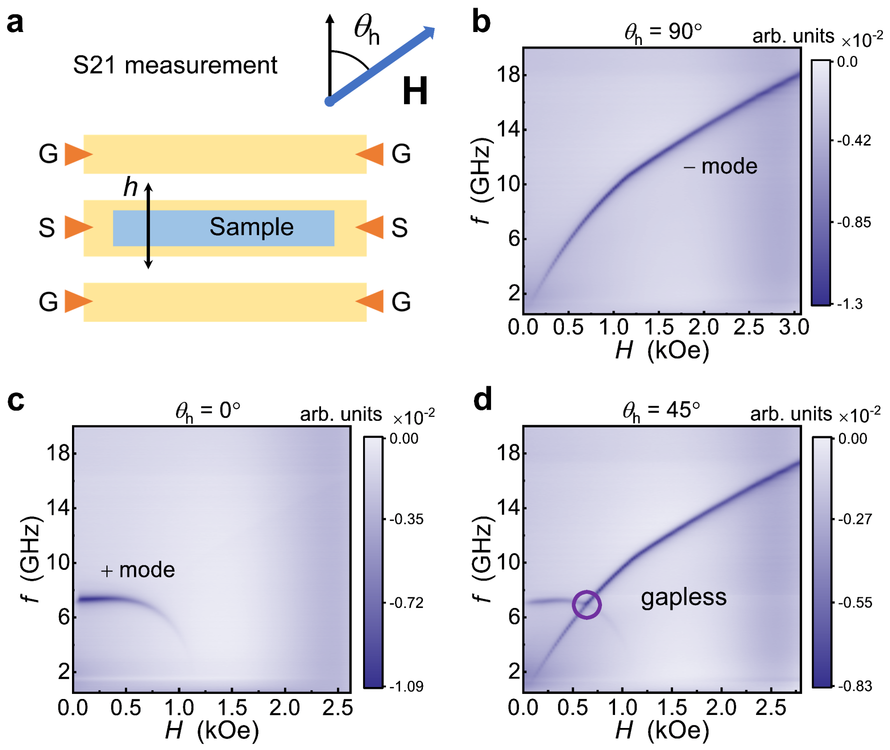

Fig. S3.

Measured resonance spectra of the reference sample. a Schematic of vector network analyzer ferromagnetic resonance (VNA FMR) measurement. A coplanar waveguide is patterned on the sample. And an external magnetic field **H** is applied in the sample plane, with an angle of θ_h to the rf magnetic field *h*. **b** to **d** Measured resonance spectra of the reference sample under different field geometries, θ_h = 90°, 0° and 45° for (**b**), (**c**) and (**d**), respectively. In the field geometries of (**b**) and (**c**), only − mode and + mode can be excited, respectively. While in the field geometry of (**d**), both modes are excited. In (**d**), no gap is observed, as shown by the purple circle, indicating that the − and + modes in the reference sample are decoupled.

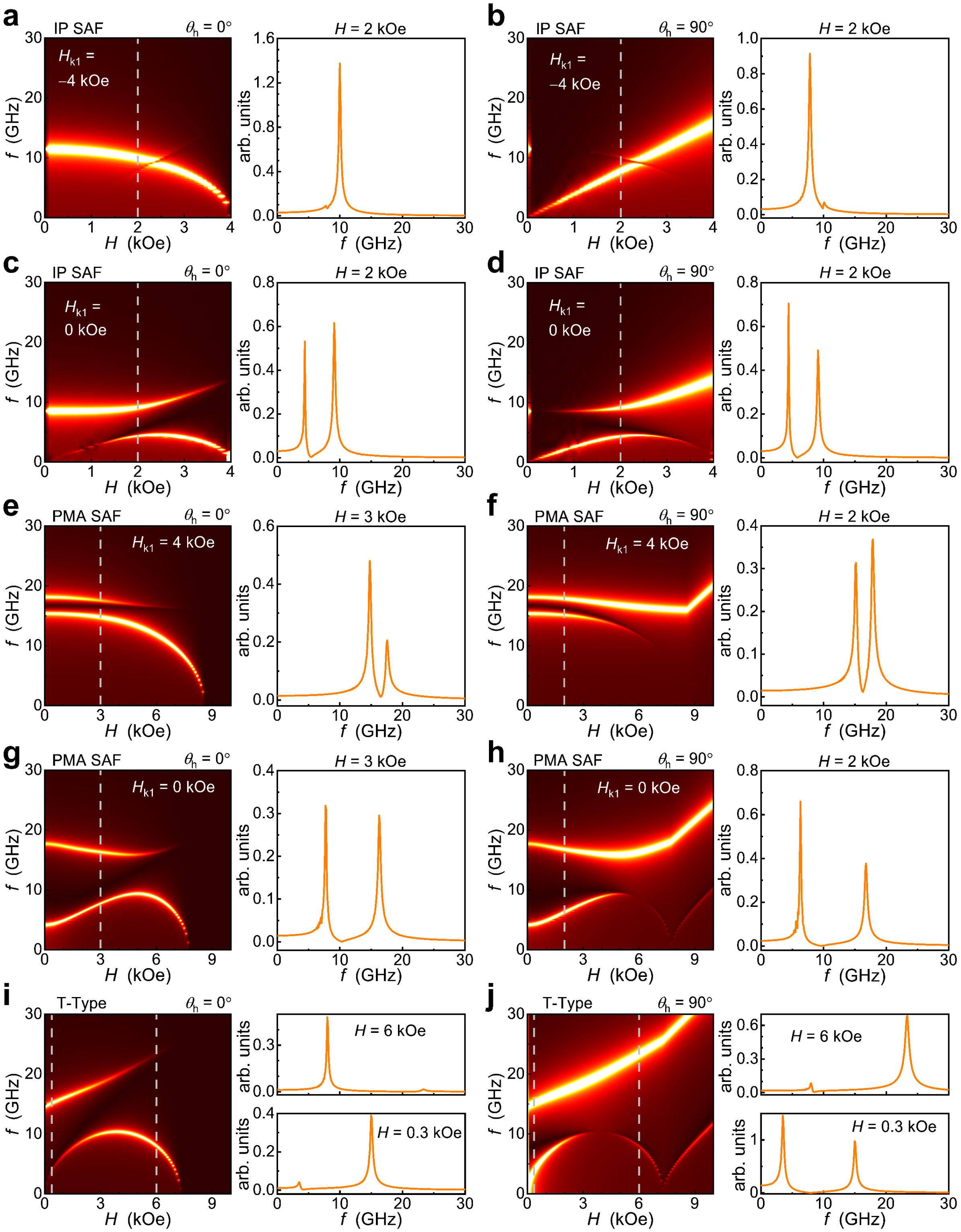
a
IP SAF
θ_h = 0°
H_{k1} = −4 kOe
H = 2 kOe
b
IP SAF
θ_h = 90°
H_{k1} = −4 kOe
H = 2 kOe
c
IP SAF
θ_h = 0°
H_{k1} = 0 kOe
H = 2 kOe
d
IP SAF
θ_h = 90°
H_{k1} = 0 kOe
H = 2 kOe
e
PMA SAF
θ_h = 0°
H_{k1} = 4 kOe
H = 3 kOe
f
PMA SAF
θ_h = 90°
H_{k1} = 4 kOe
H = 2 kOe
g
PMA SAF
θ_h = 0°
H_{k1} = 0 kOe
H = 3 kOe
h
PMA SAF
θ_h = 90°
H_{k1} = 0 kOe
H = 2 kOe
i
T-Type
θ_h = 0°
H = 6 kOe
H = 0.3 kOe
j
T-Type
θ_h = 90°
H = 6 kOe
H = 0.3 kOe
f (GHz)
H (kOe)
arb. units

Fig. S4.

Typical examples of simulated resonance spectra under different field geometries. a to **d** Two examples of IP SAF with relatively small (**a** and **b**) and large (**c** and **d**) magnetic anisotropic asymmetries. (**a** and **c**) correspond to the case of $\theta_h = 0°$, and (**b** and **d**) correspond to the case of $\theta_h = 90°$, where θ_h is defined the same as in Fig. S3. For these two IP SAF cases, they share the same H_{k2} (−5 kOe) and H_{ex} (−2 kOe), but have different H_{k1}. In (**a** and **b**), $H_{k1} = -4$ kOe, and in (**c** and **d**), $H_{k1} = 0$ kOe. **e** to **h** Two examples of PMA SAF with relatively small (**e** and **f**) and large (**g** and **h**) magnetic anisotropic asymmetries. Similarly, (**e** and **g**) and (**f** and **h**) correspond to the case of $\theta_h = 0°$ and 90°, respectively. For these two PMA SAF cases, they share the same H_{k2} (5 kOe) and H_{ex} (−2 kOe), but have different H_{k1}: 4 kOe for (**e** and **f**), and 0 kOe for (**g** and **h**). **i** and **j** An example of T-Type, where (**i**) and (**j**) correspond to the case of $\theta_h = 0°$ and 90°, respectively. In this T-Type case, $H_{k1} = -10$ kOe, $H_{k2} = 5$ kOe and $H_{ex} = -2$ kOe. In each plot, the panel(s) on the right represent(s) the corresponding curve(s) plotted along the dashed segment(s) on the left panel.

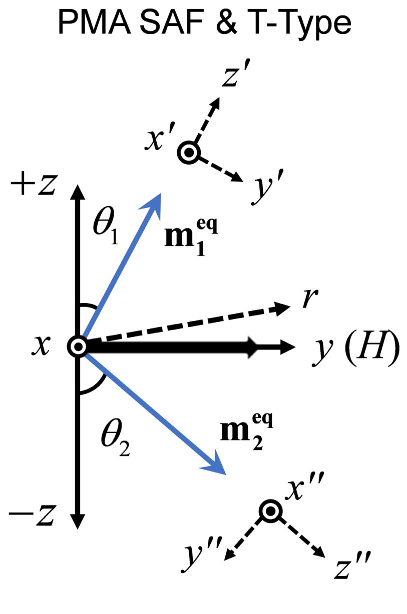

Fig. S5.

The coordinate systems used in Section S5. The one on the left is for PMA SAF (T-Type), and the one on the right is for IP SAF.

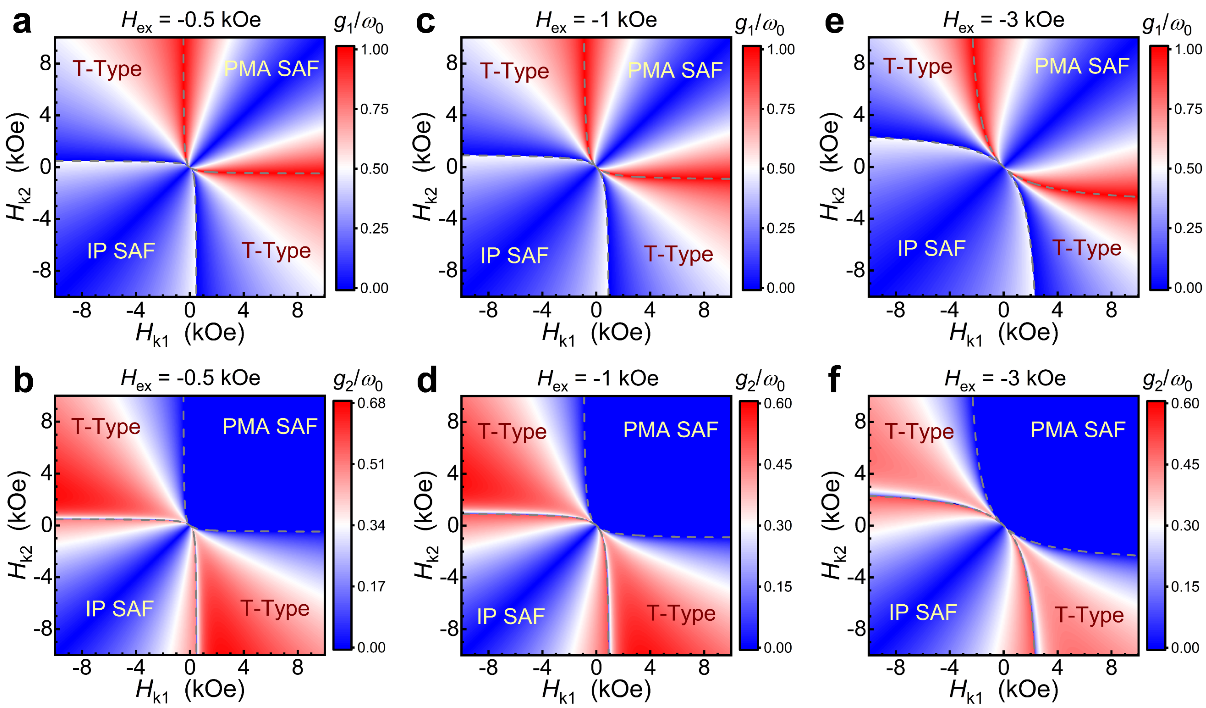

Fig. S6.

Calculated normalized coupling strength $g_{1(2)}/\omega_0$ as a function of H_{k1} and H_{k2} with different RKKY interaction strengths. a to **f** Color plots of calculated g_1/ω_0 (**a**, **c** and **e**) and g_2/ω_0 (**b**, **d** and **f**) as functions of H_{k1} and H_{k2} when H_{ex} is set to –0.5 kOe (**a** and **b**), –1 kOe (**c** and **d**) and –3 kOe (**e** and **f**). Grey dashed curves correspond to the boundary between two different magnetization configurations in each case.

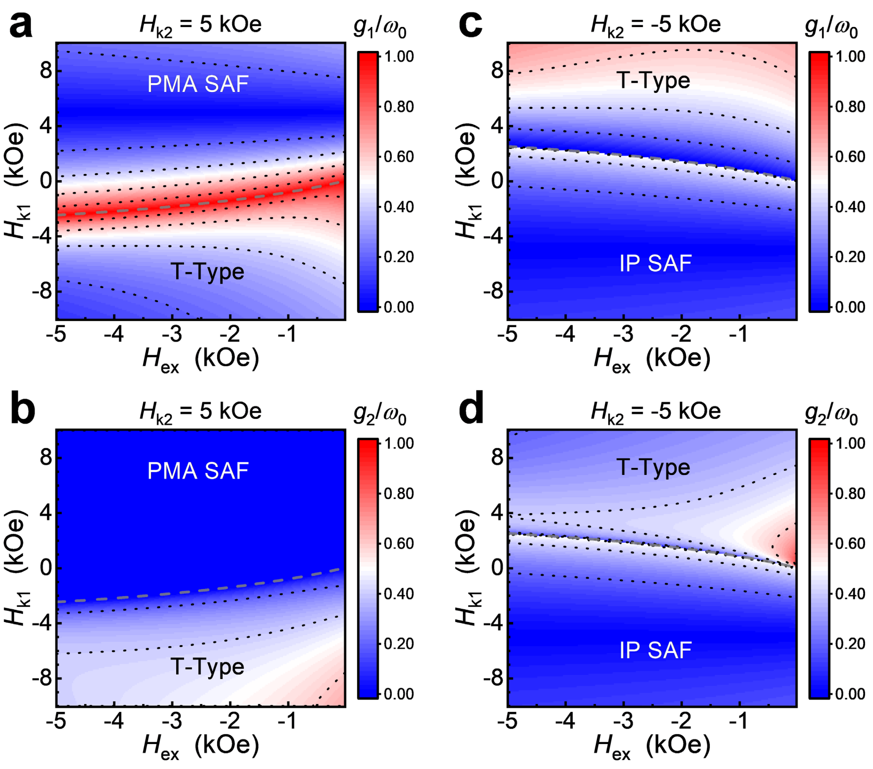

Fig. S7.

Calculated $g_{1(2)}/\omega_0$ as a function of H_{k1} and H_{ex} in different configuration regions. a to **d** Contour plots of g_1/ω_0 (**a** and **c**), g_2/ω_0 (**b** and **d**) as functions of H_{k1} and H_{ex}. H_{k2} is set to 5 kOe (**a** and **b**) and −5 kOe (**c** and **d**). Grey dashed curve in each plot indicates the boundary between two magnetization configurations.

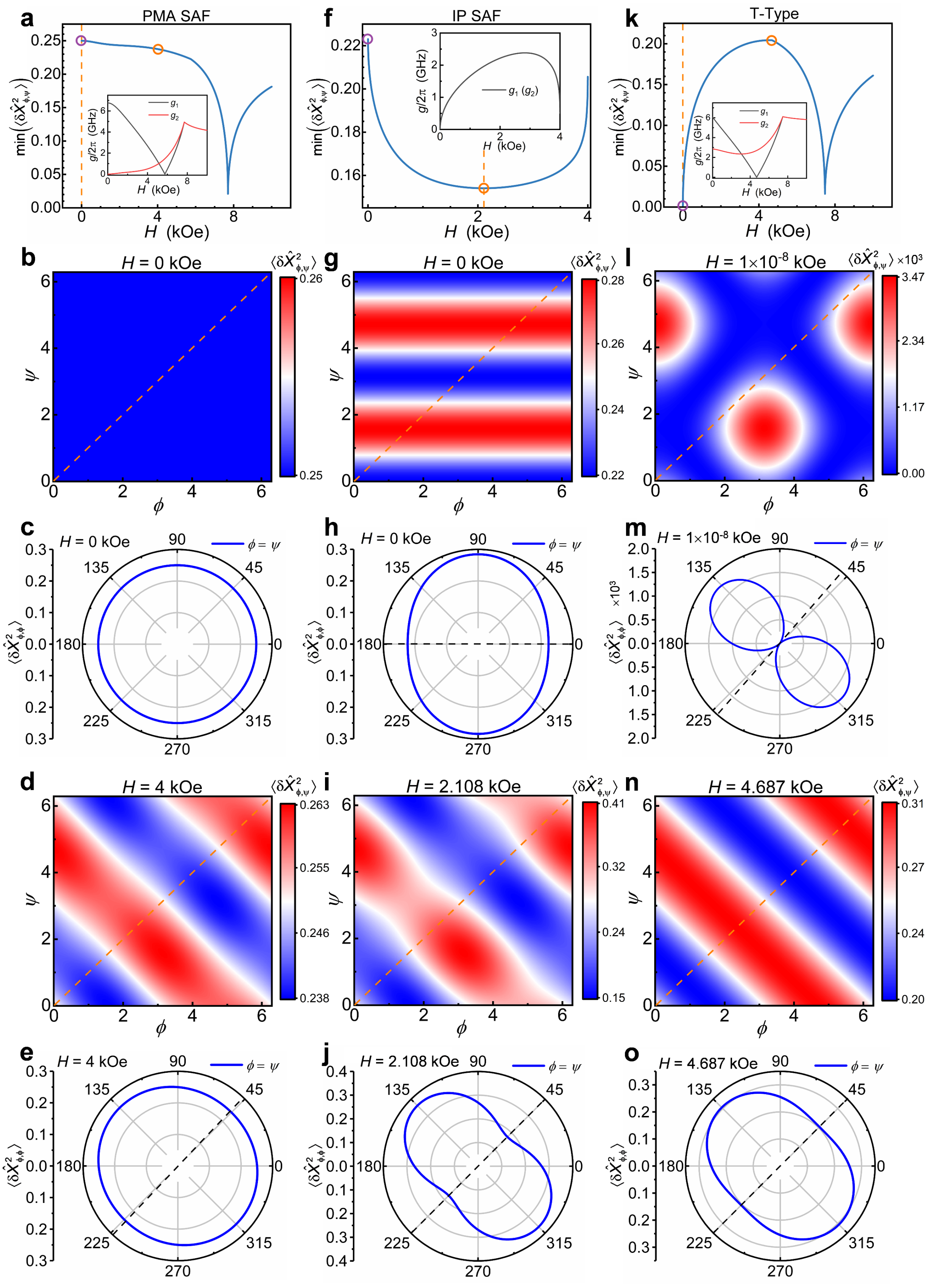

Fig. S8.

Typical examples of calculated quantum fluctuations in the ground states of SAFs tuned by *H*. **a** to **o** Quantum fluctuations for the three SAF configurations. (**a** to **e**) correspond to a typical PMA SAF configuration where H_{k1} = 0 kOe, H_{k2} = 5 kOe. (**f** to **j**) correspond to a typical IP SAF configuration where H_{k1} = 0 kOe, H_{k2} = –5 kOe. And (**k** to **o**) correspond to a typical T-Type configuration where H_{k1} = –3 kOe, H_{k2} = 5 kOe. (**a**, **f** and **k**) Minimum quantum fluctuations as a function of H. The orange dashed line in each plot indicates the corresponding magnetic field H_0. The insets display the coupling strengths g_1 and g_2 of these three structures as functions of external magnetic field H. Violet and orange circles emphasize two typical cases in each structure, shown in (**b**, **c**, **g**, **h**, **l** and **m**) and (**d**, **e**, **i**, **j**, **n** and **o**), respectively. (**b**, **d**, **g**, **i**, **l** and **n**) correspond to color plots of quantum fluctuations as functions of phase ψ and ϕ. The orange dashed line in each plot indicate the case where $\psi = \phi$. (**c**, **e**, **h**, **j**, **m** and **o**) more clearly show the relationship between quantum fluctuations and ψ (ϕ) in this case. Grey dashed line labels the minimum quantum fluctuation in each plot.

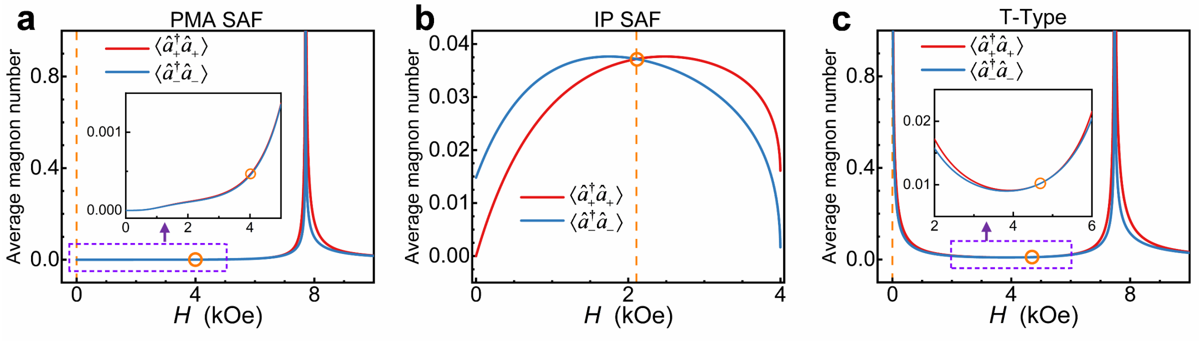

Fig. S9.

Typical examples of calculated average magnon numbers in the ground states of SAFs tuned by *H*. a to **c** Calculated average + mode magnon numbers $\left\langle \hat{a}_+^\dagger \hat{a}_+ \right\rangle$ and − mode magnon numbers $\left\langle \hat{a}_-^\dagger \hat{a}_- \right\rangle$ for three typical structures, including PMA SAF (**a**), IP SAF (**b**) and T-Type (**c**). The parameters of these SAFs are set to the same as that in Fig. S8. Insets in (**a**) and (**c**) zoom in on the regions of the violet rectangles. And the orange circles indicate the same *H* as the orange circles in Figs. S8a, S8f and S8k.

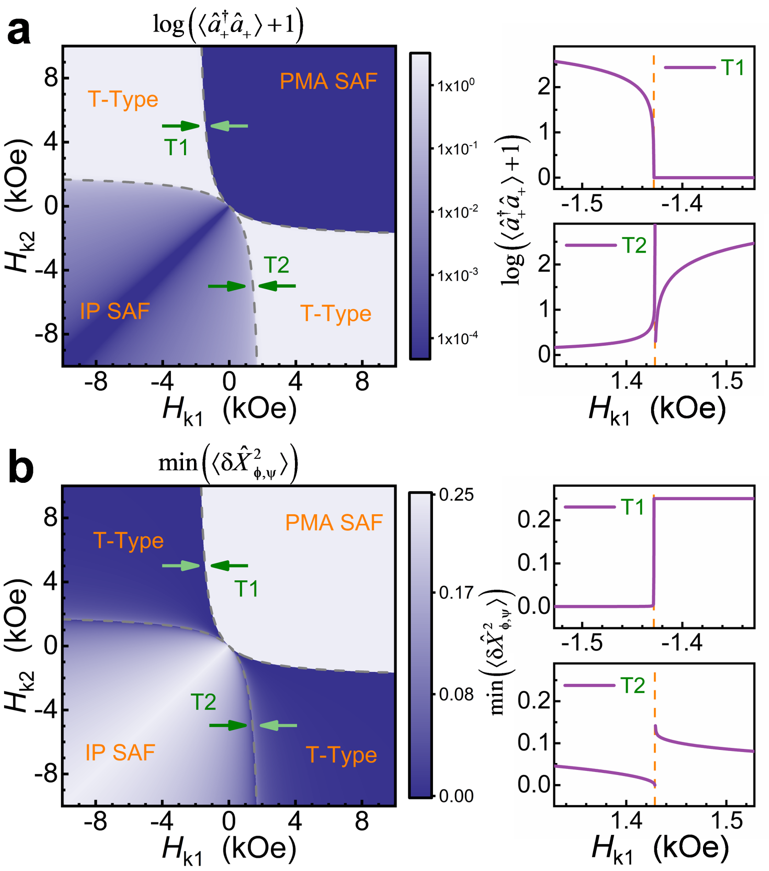

Fig. S10.

Calculated squeezed quantum fluctuations and average magnon numbers in the ground states of SAFs. a Color plots of calculated average + mode magnon number at H_0 as functions of H_{k1} and H_{k2}. $\log\left(\left\langle \hat{a}_+^\dagger \hat{a}_+ \right\rangle + 1\right)$ is displayed for clarity. Grey dashed curves in each plot indicate the boundary between two different magnetization configurations. In T-Type, a slight 10^{-5} Oe deviation from H_0 is considered to avoid divergence. We use green arrows labeled T1 and T2 to emphasize the cases near the boundary, which are shown on the right in detail. Orange dashed lines indicate the corresponding boundary. And H_{k2} is set to 5 kOe and −5 kOe for T1 case and T2 case, respectively. **b** Color plots of calculated minimum quantum fluctuation $\min\left(\left\langle \delta \hat{X}^2_{\phi,\psi} \right\rangle\right)$ at H_0 as functions of H_{k1} and H_{k2}. Still, the grey dashed curves indicate the boundary and a 10^{-5} Oe deviation from H_0 is considered in T-Type. Similarly, the results near the boundary are shown on the right in detail.

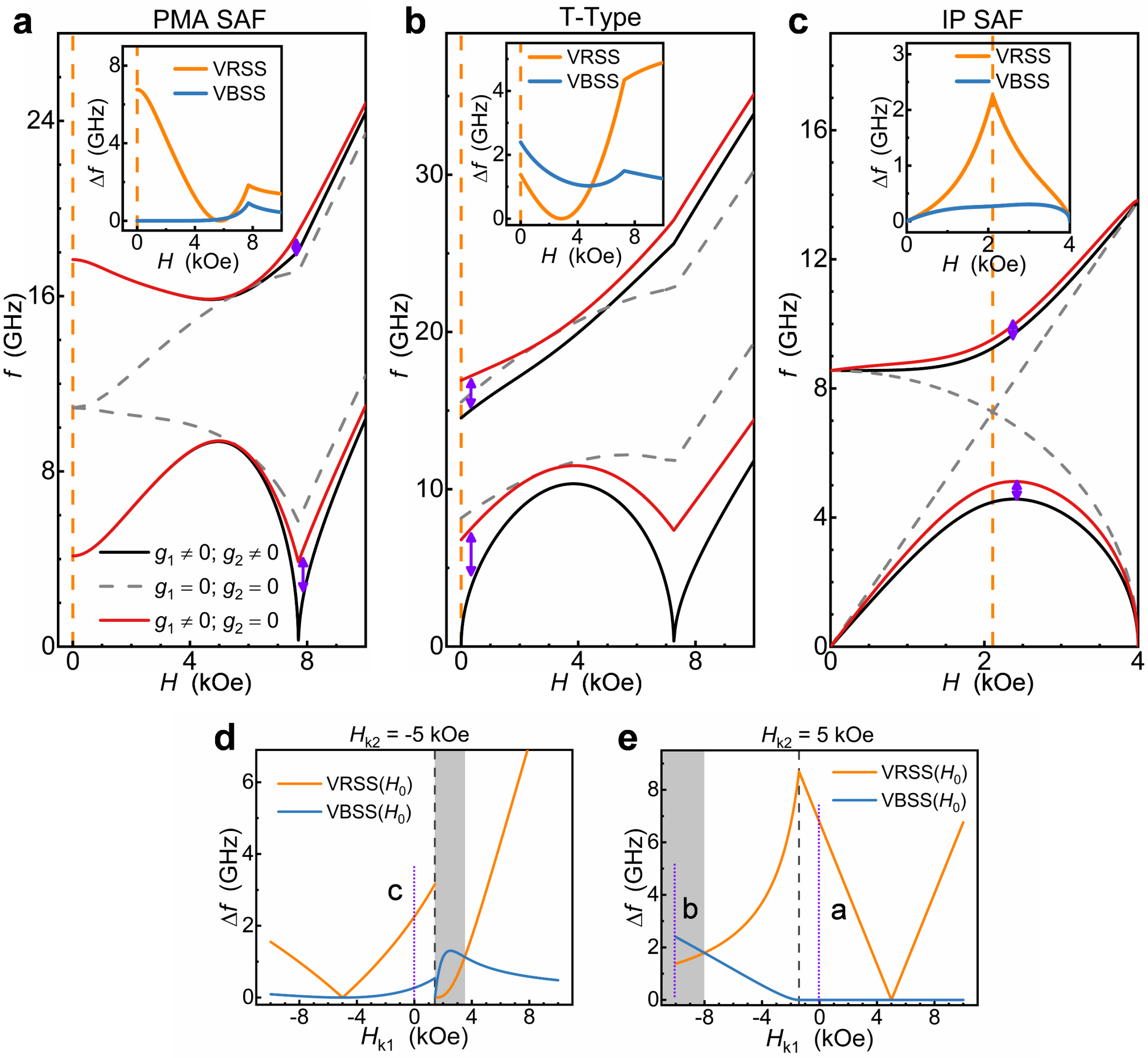

Fig. S11.

Calculated vacuum Rabi splitting-induced shifts (VRSSs) and vacuum Bloch-Siegert shifts (VBSSs). a to **c** Typical examples of calculated branch I and branch II (black solid curves), the decoupled − and + modes (gray dashed curves) and the co-rotating coupled branches where g_2 = 0 (red solid curves). (H_{k1}, H_{k2}) are set to (0 kOe, 5 kOe) (**a**), (−10 kOe, 5 kOe) (**b**) and (0 kOe, −5 kOe) (**c**). The VRSSs are indicated by the frequency differences between red solid curves and gray dashed curves, while the VBSSs are indicated by the frequency differences between red solid curves and black solid curves, which are labelled by the violet arrows. The inset in each plot displays the corresponding extracted VRSS and VBSS of branch I. Orange dashed lines in each plot and each inset indicate the magnetic field H_0 corresponding to the center frequency ω_0. **d** and **e** The VRSS(H_0) and VBSS(H_0) as functions of H_{k1} when H_{k2} is fixed at −5 kOe (**d**) and 5

kOe (**e**). Violet dashed lines indicate the cases in (**a**), (**b**) and (**c**), marked by a, b and c, respectively. The gray dashed line in each plot represents the boundary between the two regions with different magnetization configurations. And the gray color marks the regions where VBSS(H_0) > VRSS(H_0).

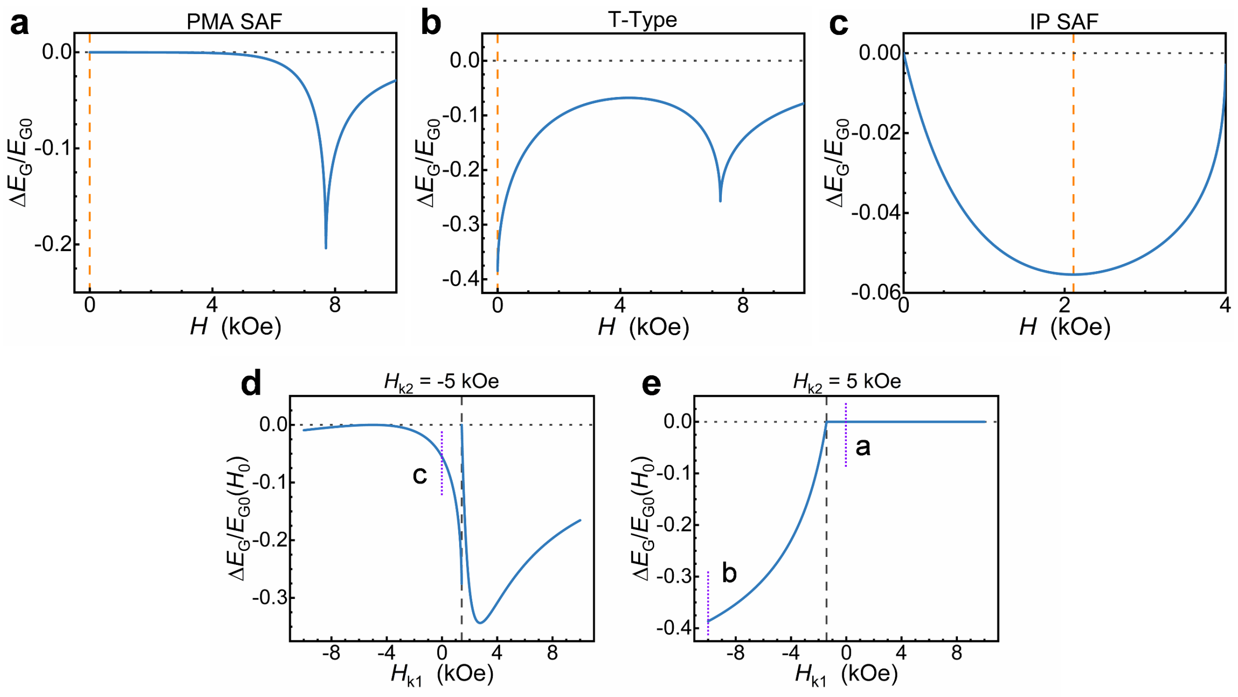

Fig. S12.

Calculated ground-state energy. a to **c** Typical examples of calculated ground-state energy variation as a function of H. From (**a**) to (**c**), (H_{k1}, H_{k2}) are the same as that in Fig. S11. The horizontal dotted line in each plot serves as a reference, indicating zero variation compared with the uncoupled case. **d** and **e** The ground-state energy variation $\Delta E_G/E_{G0}$ at H_0 as a function of H_{k1} when H_{k2} is fixed at −5 kOe (**d**) and 5 kOe (**e**). Similarly, horizontal dotted line in each plot serves as a reference. And the violet dashed lines indicate the cases in (**a**), (**b**) and (**c**), marked by a, b and c, respectively.

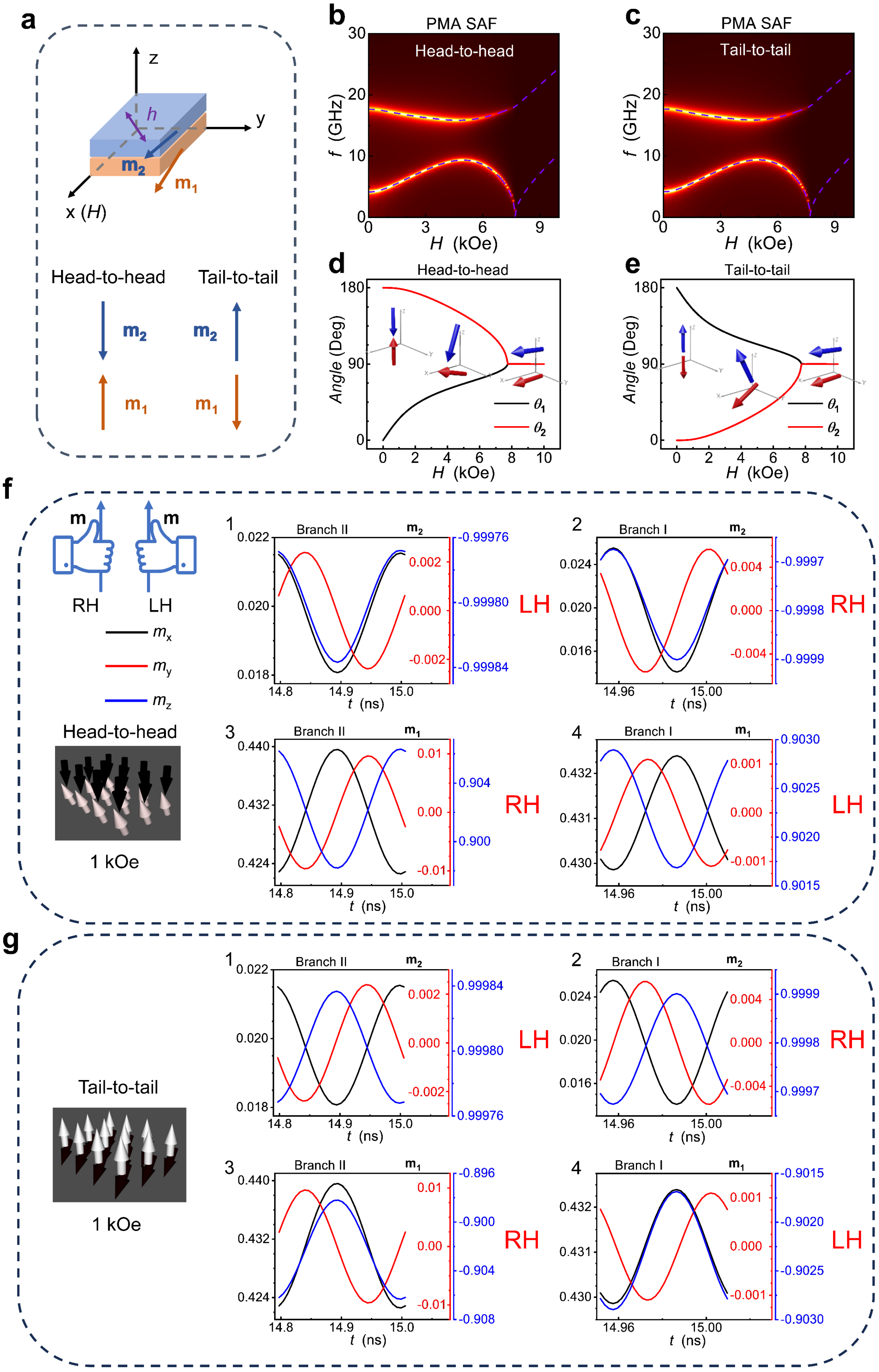
a
z
y
x (H)
h
m2
m1
Head-to-head
Tail-to-tail
b
PMA SAF
Head-to-head
f (GHz)
H (kOe)
c
PMA SAF
Tail-to-tail
d
Head-to-head
Angle (Deg)
θ1
θ2
e
Tail-to-tail
f
RH
LH
mx
my
mz
Head-to-head
1 kOe
Branch II
Branch I
t (ns)
g
Tail-to-tail
1 kOe

Fig. S13.

Comparison of SAFs with different initial configurations. a Schematic of micromagnetic simulation configuration. External magnetic field **H** is applied in the +x axis. And the rf magnetic field is applied in the SAF plane at an angle of 45° to the +x axis. Two initial configurations: "head-to-head" configuration and "tail-to-tail" configuration, are considered in the PMA SAF structure. **b** and **c** Calculated and simulated resonance spectra of a typical PMA SAF example with head-to-head configuration (**b**) and tail-to-tail configuration (**c**). The parameters of this PMA SAF example are: H_{k1} = 0 kOe, H_{k2} = 5 kOe, and H_{ex} = −2 kOe. The color plots show the resonance spectra obtained from micromagnetic simulation, and the violet dashed curves show the resonance spectra obtained from the theoretical calculation. **d** and **e** The polar angle $\theta_{1(2)}$ as a function of H obtained from micromagnetic simulation, where (**d**) and (**e**) correspond to head-to-head configuration and tail-to-tail configuration, respectively. The insets in each plot show the schematics of equilibrium positions of the magnetic moments $\mathbf{m_1}$ and $\mathbf{m_2}$ at different values of H. **f** and **g** Simulated magnetization oscillations of $\mathbf{m_1}$ and $\mathbf{m_2}$ when the initial configurations are head-to-head (**f**) and tail-to-tail (**g**). H is set to 1 kOe during the micromagnetic simulation. The schematics of the right-hand (RH) polarization and left-hand (LH) polarization of $\mathbf{m_1}$ and $\mathbf{m_2}$ are shown in (**f**). Panels 1 to 4 in each plot show the oscillations of the components of the magnetization unit vector m_x, m_y and m_z in the time domain, where Panels 1 and 3 correspond to the oscillations of $\mathbf{m_1}$ (Panel 3) and $\mathbf{m_2}$ (Panel 1) when the branch II is excited, and Panels 2 and 4 correspond to the oscillations of $\mathbf{m_1}$ (Panel 4) and $\mathbf{m_2}$ (Panel 2) when the branch I is excited. The polarization of $\mathbf{m_{1(2)}}$ in each panel is indicated on the right.

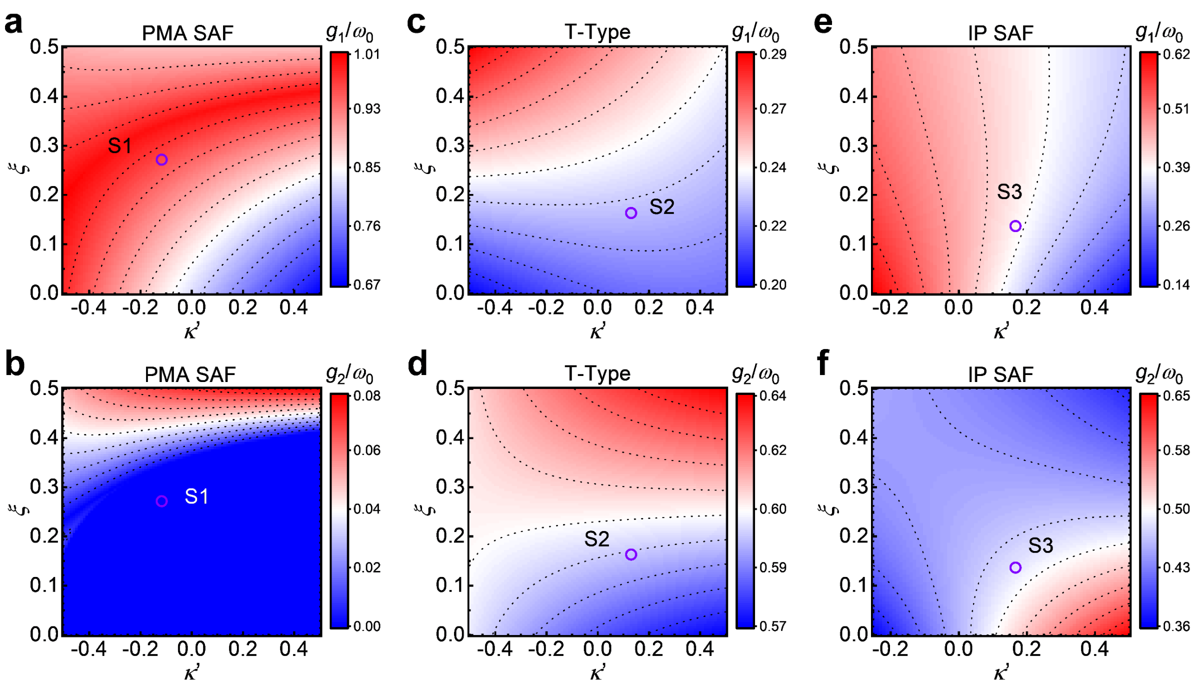

Fig. S14.

Effect of the biquadratic exchange interaction and asymmetry of the saturation magnetization *M*S and thickness *d* between FM1 and FM2 on coupling properties. Color plots of the g_1/ω_0 and g_2/ω_0 as functions of ξ and κ' calculated based on the complete case. ξ represents the ratio of the biquadratic exchange field to the bilinear exchange field, and κ' is the factor used to quantify the asymmetry of M_S and d. (**a** and **b**), (**c** and **d**) and (**e** and **f**) correspond to the results of PMA SAF, T-Type and IP SAF, respectively. In these cases, H_{k1}, H_{k2} and $\overline{H}_{ex1} = (H_{ex1}^{(1)} + H_{ex1}^{(2)})/2$ are set to the same as the samples S1, S2 and S3, respectively. Violet circle in each plot indicates the measured (κ', ξ) of the corresponding sample.

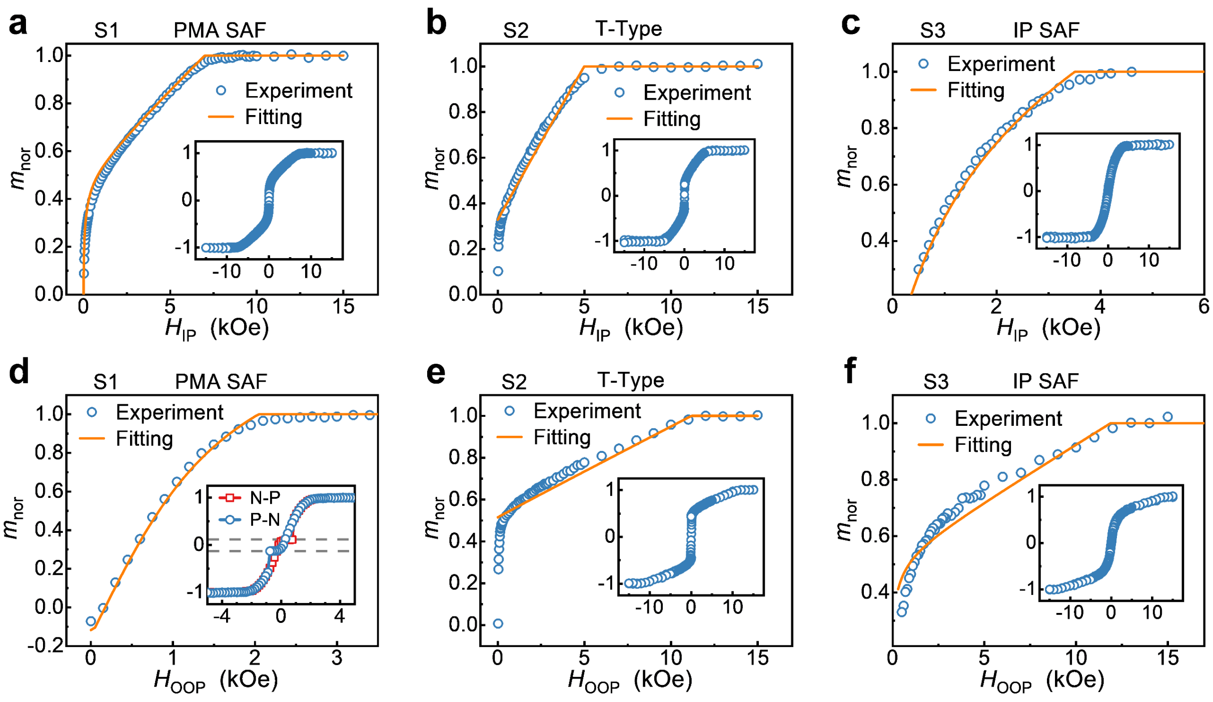

Fig. S15.

Normalized hysteresis loops measured by the vibrating sample magnetometry (VSM). (**a** to **c**) correspond to the results of samples S1, S2 and S3 measured with in plane *H*, respectively. (**d** to **f**) correspond to the results of samples S1, S2 and S3 measured with out of plane *H*, respectively. Orange curve in each plot corresponds to the fitting result based on the macrospin approach. Insets represent full scale normalized hysteresis loops. The inset in (**d**) utilizes two different colors to represent two processes, where the magnetic field sweeps from negative (N) to positive (P) and from P to N, respectively. Two platforms marked by the horizontal dashed lines are observed, confirming that S1 is a PMA SAF configuration.

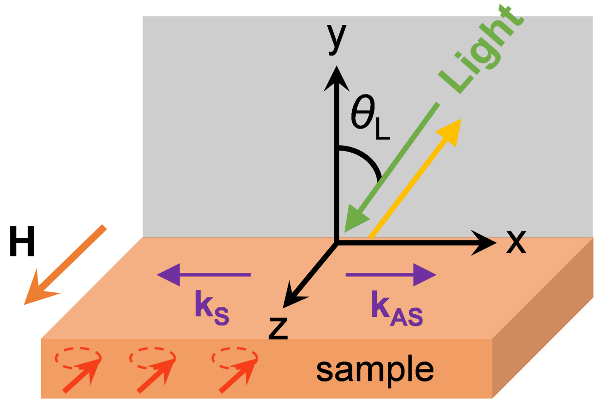

Fig. S16.

Schematic of the Brillouin light scattering (BLS) measurement. External magnetic field **H** is applied along the +*z* axis. And the light is perpendicular to **H**, with an angle of θ_L to the +*y* axis.

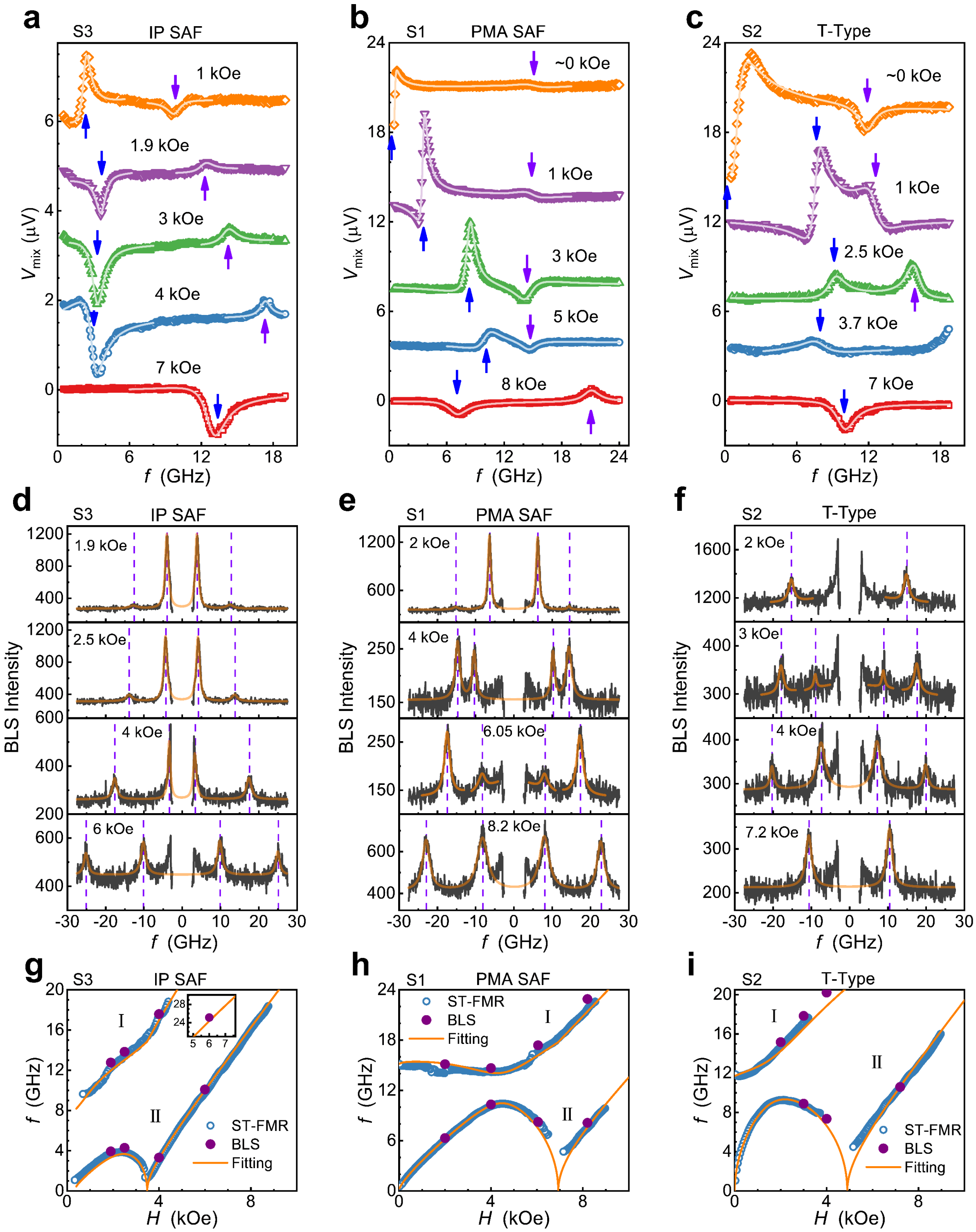

a
S3
IP SAF
1 kOe
1.9 kOe
3 kOe
4 kOe
7 kOe
V_{mix} (μV)
f (GHz)
b
S1
PMA SAF
~0 kOe
1 kOe
3 kOe
5 kOe
8 kOe
c
S2
T-Type
~0 kOe
1 kOe
2.5 kOe
3.7 kOe
7 kOe
d
S3
IP SAF
1.9 kOe
2.5 kOe
4 kOe
6 kOe
BLS Intensity
e
S1
PMA SAF
2 kOe
4 kOe
6.05 kOe
8.2 kOe
f
S2
T-Type
2 kOe
3 kOe
4 kOe
7.2 kOe
g
S3
IP SAF
I
II
ST-FMR
BLS
Fitting
f (GHz)
H (kOe)
h
S1
PMA SAF
i
S2
T-Type

Fig. S17.

Details of the spectra shown in Fig. 4. a to **c** Some representative spin torque ferromagnetic resonance (ST-FMR) spectra selected from the color plots in Fig. 4. (**a**), (**b**) and (**c**) correspond to the samples S3, S1 and S2, respectively. In each plot, five representative spectra are shown. The solid curve(s) in each spectrum correspond(s) to the Lorentzian fitting result of experimental data. And the blue and violet arrows indicate the resonance frequencies $f_{\mathrm{r}}^{\mathrm{l}}$ and $f_{\mathrm{r}}^{\mathrm{h}}$, respectively. **d** to **f** BLS spectra for S3 (**d**), S1 (**e**), and S2 (**f**). The experimental data are fitted through the Lorentzian function, as shown by the orange curves. And the violet dashed lines indicate the resonance frequencies extracted from the Stokes peaks and anti-Stokes peaks. **g** to **i** Extracted resonance frequencies and the corresponding fitting results for S3 (**g**), S1 (**h**), and S2 (**i**). The blue circles correspond to the resonance frequencies extracted from the ST-FMR spectra in Fig. 4. The purple dots, including one in the inset of (**g**), correspond to the resonance frequencies extracted from the Stokes peaks in (**d** to **f**). And the orange curves correspond to the fitting results based on the complete case.

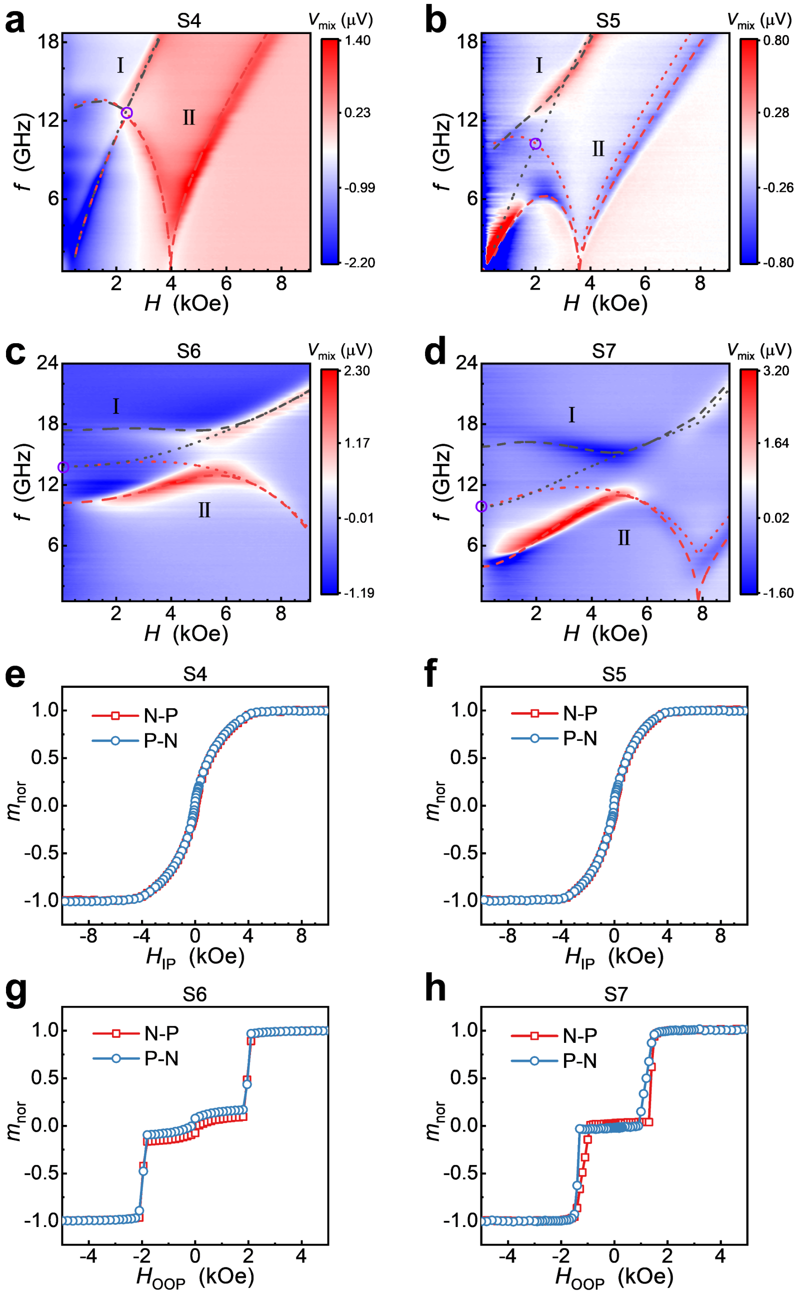

Fig. S18.

Resonance spectra and normalized hysteresis loops of samples S4 to S7. a to **d** Resonance spectra of two other IP SAF samples: S4 (**a**) and S5 (**b**), and two other PMA SAF samples: S6 (**c**) and S7 (**d**). For S4 and S5 (S6 and S7), H_{k1} (H_{k2}) is tuned by adjusting the composition and period of Co/Ni stack of FM1 (FM2). The grey and red dashed curves, the grey and red dotted curves, and the violet circles are defined the same as that in Figs. 4a, 4b and 4c. **e** to **h** Normalized hysteresis loops of S4 (**e**) and S5 (**f**) measured with in plane H, and S6 (**g**) and S7 (**h**)

measured with out of plane *H*. In each plot, the red curve is obtained by sweeping *H* from negative to positive value while the blue curve is obtained by sweeping *H* from positive to negative value. These hysteresis loops prove the IP SAF magnetization configuration of S4 and S5 and the PMA SAF magnetization configuration of S6 and S7.

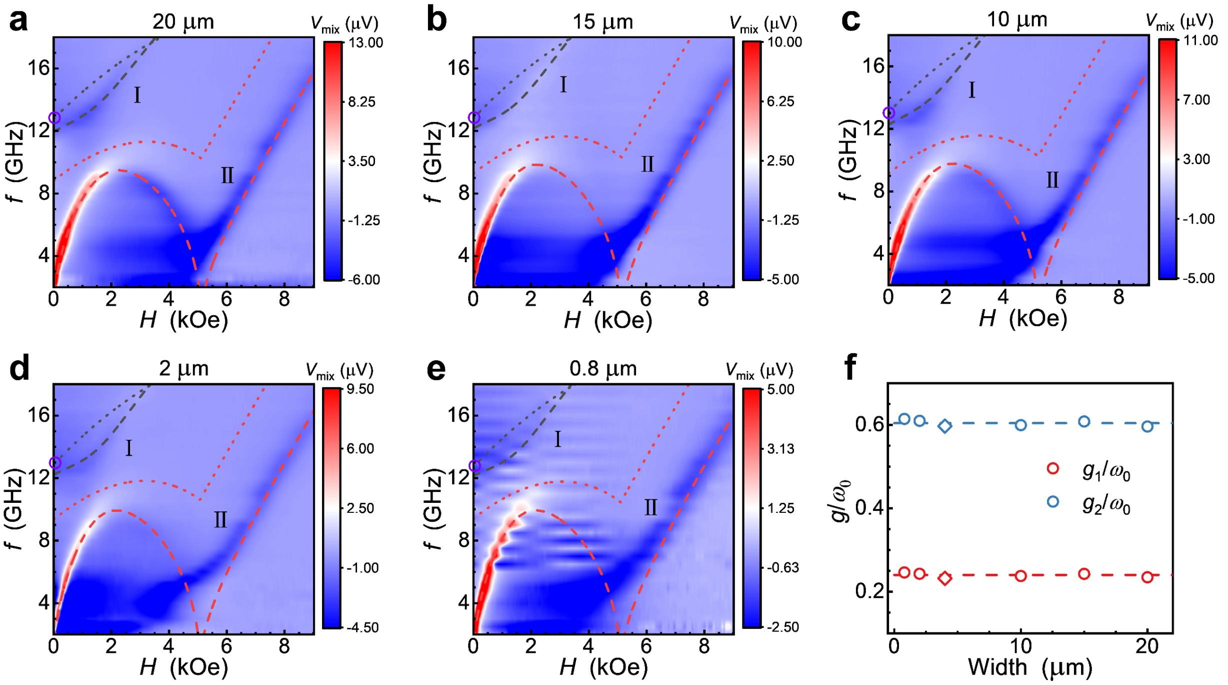

Fig. S19.

Demonstration of the effect of sample volume on the coupling strength. a to **e** Resonance spectra of sample S2 with different strip widths. From (**a**) to (**e**), the strips are scaled down from 20 μm to 0.8 μm in width. **f** Extracted g_1/ω_0 (red circles) and g_2/ω_0 (blue circles) as functions of strip width. The red and blue diamonds represent the g_1/ω_0 and g_2/ω_0 of S2 obtained in the main text, respectively. Red and blue horizontal dashed lines correspond to the fitting result.

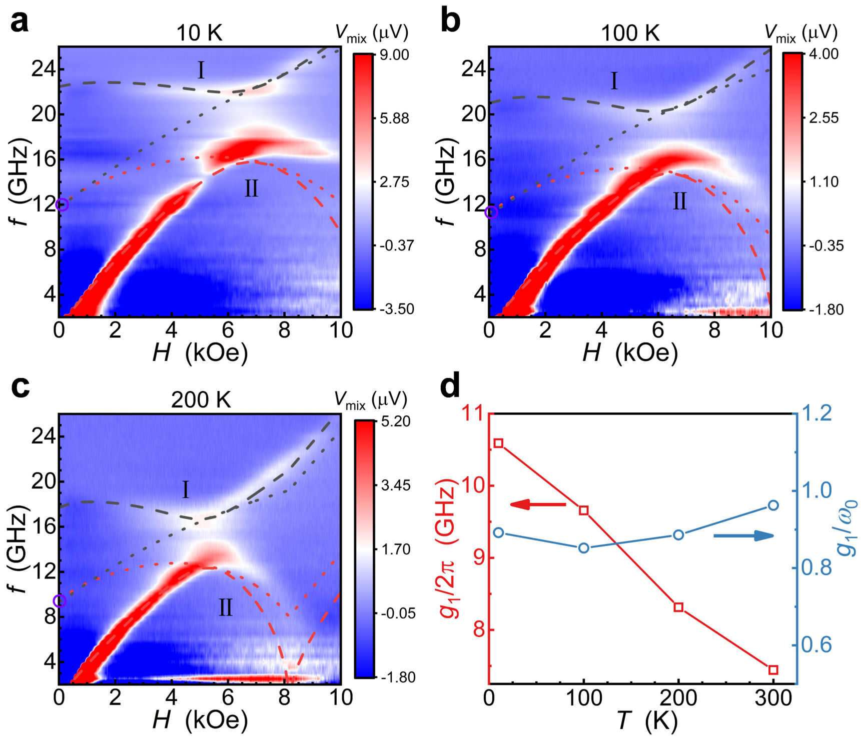

Fig. S20.

Effect of temperature on the coupling strength. a to **c** The measured resonance spectra of sample S1 at different temperatures. From (**a**) to (**c**), the temperature is set to 10 K, 100 K, and 200 K, respectively. **d** The extracted $g_1/2\pi(H_0)$ (red squares) and g_1/ω_0 (blue circles) as functions of temperature T.

Table S1.

SAF Parameters extracted from hysteresis loops and resonance spectra for the studied samples.

Parameters	S1	S2	S3
H_{k1} (Oe)	4900	3720	750
H_{k2} (Oe)	−250	−9570	−9570
$H_{ex1}^{(2)} / H_{ex1}^{(1)}$	0.79	1.30	1.40
Hysteresis loops:			
$H_{ex1}^{(1)}$ (Oe)	−1050	−800	−1080
$H_{ex2}^{(1)}$ (Oe)	−330	−170	−190
g_1/ω_0	0.987	0.237	0.402
g_2/ω_0	0	0.602	0.484
Resonance spectra:			
$H_{ex1}^{(1)}$ (Oe)	−1074	−803	−1140
$H_{ex2}^{(1)}$ (Oe)	−292	−131	−156
g_1/ω_0	0.963	0.232	0.392
g_2/ω_0	0	0.597	0.487

Table S2.

VRSSs(H_0), VBSSs(H_0), $\Delta E_G/E_{G0}(H_0)$, minimum quantum fluctuations and average + mode magnon numbers in the ground states of the studied samples.

Sample no.	VRSS(H_0) (GHz)	VBSS(H_0) (GHz)	$\Delta E_G/E_{G0}(H_0)$	$\min\left(\langle \delta\hat{X}^2_{\phi,\psi}\rangle\right)$	$\langle \hat{a}^\dagger_+ \hat{a}_+ \rangle$
S1	7.44	0	0	0.25	0
S2	1.55	2.42	–0.45	4.0×10^{-3}	2.6×10^{3}
S3	3.61	0.81	–0.17	8.3×10^{-2}	0.18
S4	0.39	0.11	-9.3×10^{-3}	0.22	4.7×10^{-3}
S5	3.07	0.57	-8.3×10^{-2}	0.14	5.8×10^{-2}
S6	3.59	0	0	0.25	0
S7	5.93	0	0	0.25	0
